# Detecting intratumoral heterogeneity of EGFR activity by liposome-based *in vivo* transfection of a fluorescent biosensor

**DOI:** 10.1038/onc.2016.522

**Published:** 2017-02-06

**Authors:** G Weitsman, N J Mitchell, R Evans, A Cheung, T L Kalber, R Bofinger, G O Fruhwirth, M Keppler, Z V F Wright, P R Barber, P Gordon, T de Koning, W Wulaningsih, K Sander, B Vojnovic, S Ameer-Beg, M Lythgoe, J N Arnold, E Årstad, F Festy, H C Hailes, A B Tabor, T Ng

**Affiliations:** 1 Richard Dimbleby Department of Cancer Research, Randall Division & Division of Cancer Studies, Kings College London, Guy’s Medical School Campus, London, UK; 2 Department of Chemistry, University College London, London, UK; 3 Breast Cancer Now Research Unit, King’s College London, London, UK; 4 UCL Centre for Advanced Biomedical Imaging, Division of Medicine, University College London, London, UK; 5 Gray Laboratories, Department of Oncology, Cancer Research UK and Medical Research Council Oxford Institute for Radiation Oncology, Oxford, UK; 6 Division of Cancer Studies, Kings College London, Guy’s Medical School Campus, London, UK; 7 Cancer Epidemiology Group, Division of Cancer Studies, King’s College London, London, UK; 8 Institute of Nuclear Medicine, University College London, London, UK; 9 King’s College London Dental Institute, Tissue Engineering and Biophotonics, Guy’s Hospital Campus, London, UK; 10 UCL Cancer Institute, Paul O’Gorman Building, University College London, London, UK

## Abstract

Despite decades of research in the epidermal growth factor receptor (EGFR) signalling field, and many targeted anti-cancer drugs that have been tested clinically, the success rate for these agents in the clinic is low, particularly in terms of the improvement of overall survival. Intratumoral heterogeneity is proposed as a major mechanism underlying treatment failure of these molecule-targeted agents. Here we highlight the application of fluorescence lifetime microscopy (FLIM)-based biosensing to demonstrate intratumoral heterogeneity of EGFR activity. For sensing EGFR activity in cells, we used a genetically encoded CrkII-based biosensor which undergoes conformational changes upon tyrosine-221 phosphorylation by EGFR. We transfected this biosensor into EGFR-positive tumour cells using targeted lipopolyplexes bearing EGFR-binding peptides at their surfaces. In a murine model of basal-like breast cancer, we demonstrated a significant degree of intratumoral heterogeneity in EGFR activity, as well as the pharmacodynamic effect of a radionuclide-labeled EGFR inhibitor *in situ*. Furthermore, a significant correlation between high EGFR activity in tumour cells and macrophage-tumour cell proximity was found to in part account for the intratumoral heterogeneity in EGFR activity observed. The same effect of macrophage infiltrate on EGFR activation was also seen in a colorectal cancer xenograft. In contrast, a non-small cell lung cancer xenograft expressing a constitutively active EGFR conformational mutant exhibited macrophage proximity-independent EGFR activity. Our study validates the use of this methodology to monitor therapeutic response in terms of EGFR activity. In addition, we found *iNOS* gene induction in macrophages that are cultured in tumour cell-conditioned media as well as an *iNOS* activity-dependent increase in EGFR activity in tumour cells. These findings point towards an immune microenvironment-mediated regulation that gives rise to the observed intratumoral heterogeneity of EGFR signalling activity in tumour cells *in vivo*.

## Introduction

In recent years, there has been a large number of drug failures at the Phase III and submission stages; particularly with targeted drugs that have specific molecular mechanisms of action but have relatively poor response rates in unselected patient population.^1^ For example, in breast cancer, the response rates of agents that target epidermal growth factor receptor (EGFR) vary between clinical studies, ranging from 6% to 49%, depending on the type of breast cancer, the stage of the disease and the treatment used.^2, 3, 4, 5^ To improve these poor response rates, it is essential to identify companion diagnostic tests that can select patients most likely to benefit from the drug.^6^ The companion diagnostic assays that were based on only EGFR expression *per se* (as a surrogate marker of clinical response) are now shown to be inadequate and insensitive in various tumour types.^7^ Furthermore, receptor expression level is not necessarily an indicator of its signalling activity.

**Figure 1:**
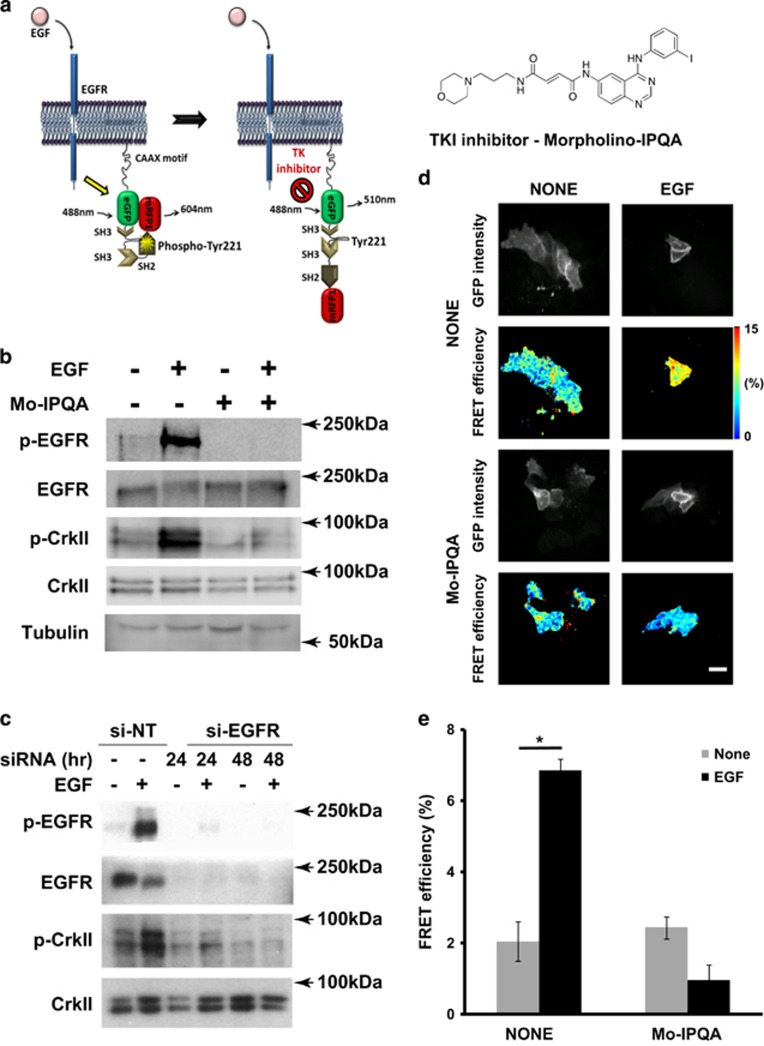
EGF-induced activation of EGFR and phosphorylation of Picchu-X. (**a**) Schematic representation of conformational changes of the Picchu-X biosensor upon EGF stimulation and inhibition with TKI enabling FRET measurement of EGFR activity. (**b**) Effect of Mo-IPQA (10 μM) on EGF-induced (100 ng/ml) EGFR activation and biosensor phosphorylation in HCC1954 cells. (**c**) Effect of EGFR knockdown on phosphorylation of biosensor. (**d**) FRET/FLIM images of cells treated as in (**b**). Scale bar is 25 μm. (**e**) Quantification of FRET efficiencies for images in (**d**). The difference between untreated and EGF-treated cells is statistically significant (data are expressed as means±s.e.m.,*P<0.002, Student’s *t*-test, *N*>10 images).

Imaging of EGFR phosphorylation as an indirect surrogate of EGFR activity has previously been achieved by determination of FRET efficiency via the method of relative donor recovery following acceptor photobleaching.^8^ The acceptor photobleaching performed was confined only to regions of interest that inevitably under-sample the tumour section. EGFR catalytic activity in cells can alternatively be directly imaged by using a FRET biosensor known as Picchu (Phosphorylation Indicator of Crk Chimeric Unit),^9^ modified to make it suitable for FRET-FLIM (Figure 1a). At present these biosensors can only be used to study murine models where the tumour has been genetically engineered to overexpress the biosensor itself.^10^ In order for the potential of these biosensors for monitoring the efficacy of drugs to be realized in a clinical setting, technology to deliver the gene coding for the biosensor selectively to tumour cells *in vivo* must be developed.

Various types of targeted nanoparticles (including liposomes) have been used successfully to deliver whole body imaging sensors and/or therapeutic agents into tumours *in vivo*^11^ due to the Enhanced Permeability and Retention effect,^12^ which is a combination of leaky tumour endothelium with wide fenestrations, in combination with a lack of effective lymphatic drainage. The efficiency of delivering the imaging sensors can be further improved by using antibody- or peptide-based^13^ approaches to target the liposomes to tumour cells that have an overexpression of EGFR.^14, 15^

Non-viral gene delivery vectors comprised of cationic lipid/DNA complexes (lipoplexes) are widely used for the *in vitro* transfection of cells, and have considerable therapeutic potential,^16^ including systemic gene delivery to tumours.^17^ However, the low efficiency of gene delivery and poor targeting ability *in vivo* have limited their development. Ternary lipid/peptide/DNA (LPD) nanoparticles (lipopolyplexes), which include receptor-targeting peptide sequences, show much higher transfection efficiencies.^18, 19^ We have developed a series of LPD nanoparticles, formulated from PEGylated lipid components designed to optimize *in vivo* nanoparticle stability, and peptide components that optimize targeting and transfection.^18, 20, 21, 22^

Peptides targeting EGFR have been developed, using phage display or *in silico* peptide library screening. GE11 (YHWYGYTPQNVI)^23^ and D4 (LARLLT)^24^ have been used to prepare EGFR-targeted liposomes,^24^ contrast agents^25^ and polyplexes.^26^ The targeting efficiency and cellular uptake of these peptides vary depending on the cell type and the nature of the bioconjugate.^25^

Here we have achieved for the first time targeted lipopolyplex delivery of an imaging biosensor that can monitor EGFR activity *in situ*, in an attempt to provide a more accurate readout of the sensitivity of tumour cells to EGFR inhibition with tyrosine kinase inhibitors (TKI) *in vivo*. This technique, coupled to a phospho-endogenous protein FLIM,^27^ shows cell-to-cell heterogeneity in EGFR activity within a basal-like breast cancer xenograft and a colorectal cancer model, which can be in part explained by a significant correlation between tumour cell EGFR activity and macrophage infiltration within the tumour microenvironment.

Furthermore, we unraveled a novel mechanism that connects the proximity of macrophages to tumour cells with the EGFR signal output and we have shown that macrophages can regulate oncogenic signalling via *iNOS* induction. Together, these findings point towards an immune microenvironment mediated regulation that gives rise to intratumoral heterogeneity of EGFR signalling activity in tumour cells.

## Results

### Imaging EGFR activity *in situ* using Picchu-FLIM

To make the EGFR activity biosensor Picchu-X^9^ compatible with high-accuracy quantitative FRET-FLIM imaging, we replaced YFP and CFP with mRFP1 and eGFP. Phosphorylation of the Y221 residue within the biosensor molecule leads to a conformational change due to interaction between pY221 and the sensor’s SH2 domain (Figure 1a), leading to decreased distance between the mRFP1 and eGFP resulting in FRET as measured by a decreased eGFP fluorescence lifetime (tau). Phosphorylation of Y221 in the Picchu-X sensor was triggered by EGFR activation following EGF stimulation (Figures 1a and c); the associated increase in FRET efficiency of the biosensor in transfected HCC1954 breast cancer cells is shown in Figure 1d (shift to ‘warmer’ colours in pseudocoloured micrographs) and Figure 1e.

The Picchu-X biosensor was further validated by EGFR knockdown (Figure 1c and Supplementary Figure S9C) and TKI treatment. We used Mo-IPQA,^28^ a second-generation version of PD168393^29^ that was developed to improve water solubility as a result of decreased lipophilicity. Mo-IPQA can be radiolabelled with radio-iodine and used for SPECT imaging *in vivo*.^26^ The effect of EGF on the phosphorylation of the biosensor was abolished by pre-treatment with Mo-IPQA (Figure 1b). Mo-IPQA at a concentration of 10 μM was effective for up to 48 h after addition (Supplementary Figure S9A) and completely abolished the phosphorylation of EGFR in EGF-stimulated HCC1954 breast cancer cells (Supplementary Figure S9B), whereas its effect was limited at a concentration of 20 μM in the MDA-MB-231 breast cancer cell line. This is likely to be due to a higher level of EGFR expression in MDA-MB-231 cells or to the cell type-specific effect of the inhibitor.^28^

### Design and optimization of EGFR targeting lipopolyplex complexes

**Figure 2:**
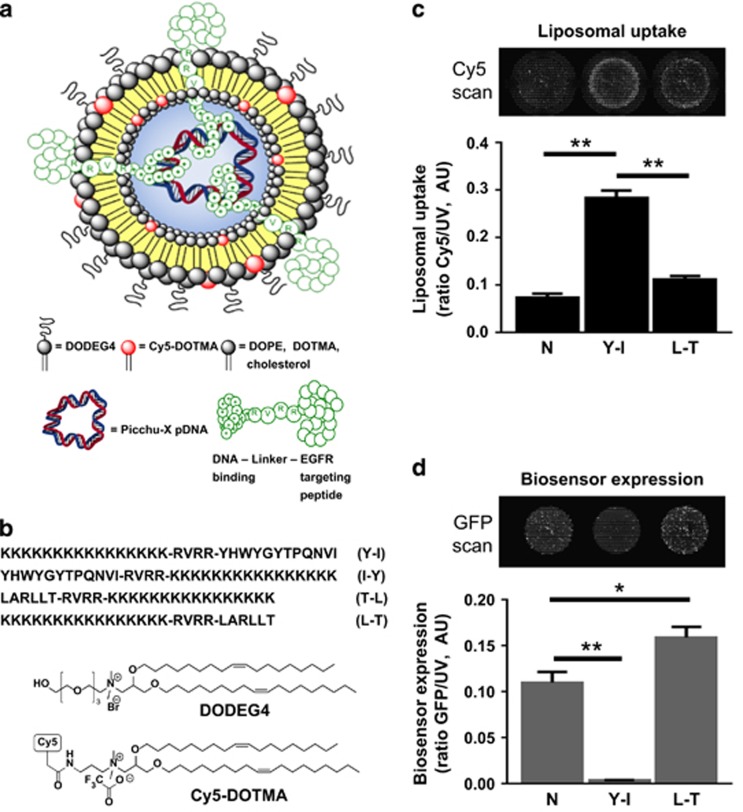
EGFR-targeted liposomal delivery system for cell transfection with the Picchu biosensor plasmid. (**a**) Schematic representation of the lipopolyplex used for targeted delivery of sensor DNA. The lipopolyplex comprises: pDNA encoding for the Picchu-X sensor; a bifunctional peptide with a cationic DNA-binding sequence and an EGFR-targeting sequence; a mixture of cationic and neutral lipids (DOPE, DOTMA, cholesterol and the PEG-shielding lipid DODEG-4); and optionally Cy-5 DOTMA. (**b**) Structures of key lipid components of the lipopolyplex and all four sequences of the bifunctional peptides used to formulate lipopolyplexes. Liposomes used in the experiments were formulated comprising: the helper lipid DOPE (23 mol%), which should fuse with the endosomal membrane and destabilize it;^16^ the cationic lipid DODEG4 (20; 39 mol%), which has a short ethylene glycol headgroup to provide steric shielding to the nanocomplex without impeding cellular uptake; DOTMA (15 mol%); and cholesterol (23 mol%). (**c**) For *in vitro* experiments, Cy5-DOTMA (1 mol%) was added to allow detection of the nanoparticles in cells. Liposome uptake (Cy5/UV ratio represents amount of liposome taken up by all cells (Hoechst staining – UV channel) in the well) in MDA-MB-231 cells treated with lipopolyplexes modified with **Y-I** or **L-T** peptides (please see Figure 2b for an explanation of the abbreviations). (**d**) Quantification of the expression of Picchu-X biosensor (eGFP/UV ratio) in the same cells as in (**c**) Complexes were prepared at 4:1 peptide/DNA weight ratio. One-way ANOVA to test differences between group means. *P* values are derived from a post-hoc Tukey’s test subsequently performed to compare means of individual groups while controlling for family-wise error rate. The difference was statistically significant between control and treatment groups, and data were expressed as means±s.e.m. (*P<0.05, **P<0.01, Student’s *t*-test, *N*=3).

**Figure 3:**
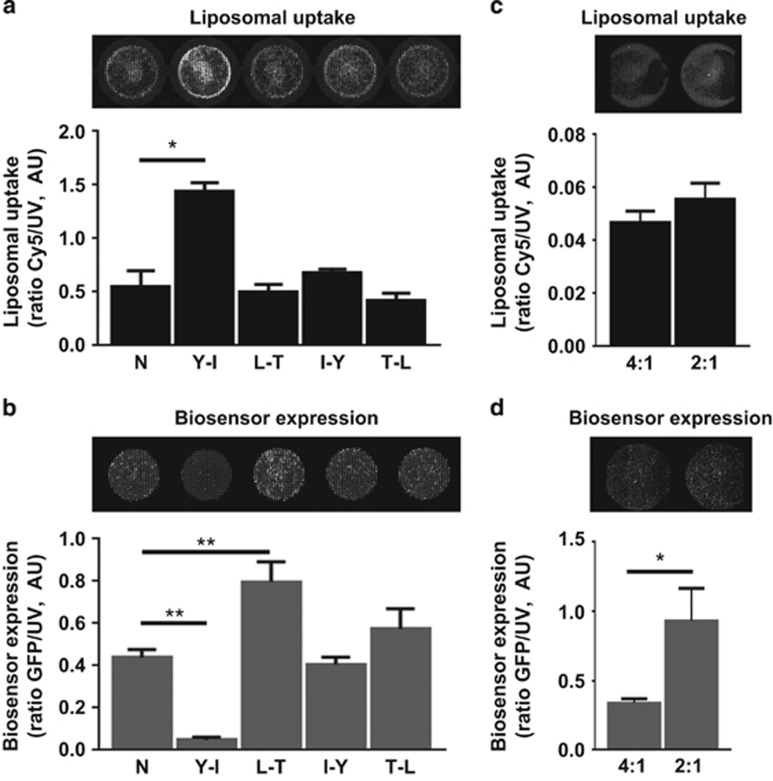
Optimization of lipopolyplexes to maximize transfection and expression of Picchu-X biosensor. (**a**) Transfection efficiency of lipopolyplexes modified with different targeting peptides in HCC1954 cells (same as in Figure 2c). (**b**) Expression of Picchu-X biosensor in the same cells treated in (**a**). (**c**, **d**). Transfection and Picchu-X biosensor expression in HCC1954 cells treated with lipopolyplexes modified with **Y-I** peptide using different peptide/DNA ratios. One-way ANOVA to test differences between group means. *P* values are derived from a post-hoc Tukey’s test subsequently performed to compare means of individual groups while controlling for family-wise error rate. The difference was statistically significant between control and treatment groups, and data were expressed as means±s.e.m. (*P<0.01, *N*=3).

In order to achieve selective delivery and transfection of the plasmid encoding the Picchu-X biosensor to tumour cells overexpressing EGFR, we designed lipopolyplexes that would display different EGFR-targeting peptides^23, 24^ at the surface of the nanoparticle (Figure 2a). Four bifunctional peptides were synthesized, containing: a linear K_16_ domain to condense the DNA; the YHWYGYTPQNVI^23^ (**Y-I**, **I-Y**) or LARLLT^24^ (**L-T, T-L**) targeting sequences; and a linker sequence (RVRR) that will be cleaved after internalization of the nanoparticle by the endosomal enzyme furin.^18, 20^ For each sequence, one peptide with the targeting moiety at the *C*-terminus **(Y-I, L-T**) and one peptide with the targeting moiety at the *N*-terminus (**I-Y, T-L**) were synthesized, in order to ascertain which would be most effective to mediate cell-selective uptake and transfection (Figure 2a). We initially tested our lipopolyplexes for their ability to deliver DNA to HCC1954 and MDA-MB-231 tumour cells and found the expected plasma membrane localization of the Picchu-X biosensor (Supplementary Figure S10B, white arrows), and different from lipid-Cy5 localization since lipopolyplexes were internalized in order to deliver the Picchu-X DNA. Although the amount of lipopolyplexes successfully delivered to MDA-MB-231 cells was ∼threefold that of non-targeting control (Cy/UV ratio, Figure 2c) when formulated using the **Y-I** peptide, the expression level of Picchu-X was reduced by comparison to non-targeting control (as monitored by the eGFP/UV ratio, Figure 2d). These results were replicated in the HCC1954 cells (Figures 3a and b, Supplementary Figure S10A). The LARLLT and YHWYGYTPQNVI targeting sequences have been shown to promote uptake of nanoparticles to tumour cells overexpressing EGFR. In our previous work^22^ we have shown that similar LPD formulations have the targeting moiety of the peptide at the surface of the lipopolyplex. We have also shown that lipopolyplexes formulated with a non-targeting K_16_ peptide, or with a peptide with a randomized targeting sequence, will have greatly reduced transfection, since the receptor-mediated uptake pathway will no longer be active.^30^ Since the **Y-I** targeting peptide gave a higher extent of lipopolyplex delivery to the tumour cells, we further optimized this complex to increase the expression of Picchu-X.

A decrease of peptide/DNA ratio from 4:1 to 2:1 did not change the delivery of the complex to the cells (Figure 3c), but significantly increased the expression levels of the biosensor (Figure 3d). This optimized formulation was thus used in subsequent *in vivo* experiments conducted with HCC1954 cells.

### Visualizing the distribution and clearance of the TKI Mo-IPQA in naive mice

**Figure 4:**
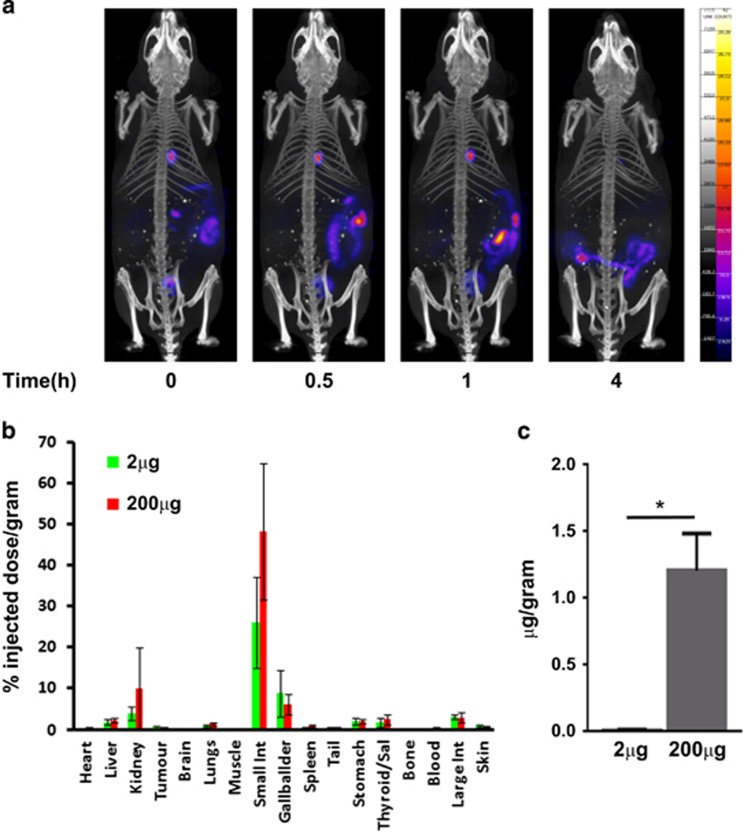
Whole body biodistribution and drug tumour uptake of ^125^I-Mo-IPQA. (**a**) SPECT/CT images of ^125^I-Mo-IPQA biodistribution throughout the whole body of a control mouse. The images show rapid uptake in the gallbladder and bladder suggesting clearance via the kidney and bile duct into the intestines. (**b**) % injected dose/gram of all tissues including tumour 1 h after either a 2-μg or 200-μg dose of Mo-IPQA doped with 0.5 Mbq (2ng) ^125^I-Mo-IPQA. (**c**) Total Mo-IPQA (equivalent % injected dose/gram) received by tumour tissue (2 μg and 200 μg). The data show a 100-fold increase in Mo-IPQA concentration within tumour tissue for the 200-μg treated mice compared to the 2 μg. There was also a small amount of uptake in the thyroid or stomach suggesting that ^125^I-Mo-IPQA is stable with little degradation of tracer resulting in free ^125^I. Tumour uptake studies were undertaken at 1 h post injection (p.i.) due to the observed rapid excretion of ^125^I-Mo-IPQA via the gallbladder and kidney in these studies.

Radioiodinated ^125^I-Mo-IPQA (see SI) was intravenously administered to naive (non-tumour bearing) mice. The whole body distribution of ^125^I-Mo-IPQA was imaged by SPECT at various time points to measure its clearance rate over a 24-h time period (Figure 4a). SPECT/CT images showed that directly after injection there was uptake in the gallbladder, small intestine and bladder, suggesting that Mo-IPQA clearance is via the kidneys into the bladder and the bile duct into the intestines (Figure 4a). I^125^Mo-IPQA uptake in the gallbladder increased between 0 and 0.5 h and was sustained at 1.5 h, but declined at 4 h. In the bladder tracer uptake remained constant, up to 1 h, indicating that the main route of excretion was via the bile duct. The ^125^I-Mo-IPQA activity within the small intestine moved towards the large intestine at the later 4-h time point indicating faecal excretion. After 24 h we were unable to detect any radioactivity by SPECT/CT imaging (data not shown). These data were aquired by SPECT/CT longitudinally in the same mice to identify an ideal time point for the quantitative measurement (% injected dose/gram) of ^125^I-Mo-IPQA uptake in a tumour model with gamma counting that is highly sensitive.

### Mo-IPQA biodistribution and tumour uptake in the breast cancer xenograft model

Biodistribution experiments in mice bearing breast cancer xenografts were performed using carrier-added ^125^I-Mo-IPQA resulting in two different doses of morpholino-IPQA (2 μg and 200 μg). The results (Figure 4b) confirmed that at 1 h p.i. the predominant uptake of ^125^I-Mo-IPQA is within the gallbladder and small intestine, confirming the results from the SPECT/CT experiment. There was also some uptake within the kidney suggesting that the main excretion route is via the bile duct into the small intestines and ultimately to the faecal matter, with some excretion via the kidney into the bladder. The tumour uptake of ^125^I-Mo-IPQA was similar for both the 2 μg and 200 μg Mo-IPQA doses, which were doped with 0.5 Mbq (2 ng) I^125^-Mo-IPQA each. The injected dose/gram values found were 0.51±0.21 and 0.49%±0.20, respectively (Figure 4b). Importantly, this translates to an approximate 100-fold difference in the tumour uptake of total Mo-IPQA in the case of the administered 200-μg dose (1.209 μg/gram±0.0534) compared to the 2-μg dose (0.012 μg/gram±0.004; *P*=0.023, *N*=4, by *t*-test; Figure 4c), demonstrating the dose-dependent tumour uptake of Mo-IPQA.

### Measurement of EGFR activity *ex vivo*

**Figure 5:**
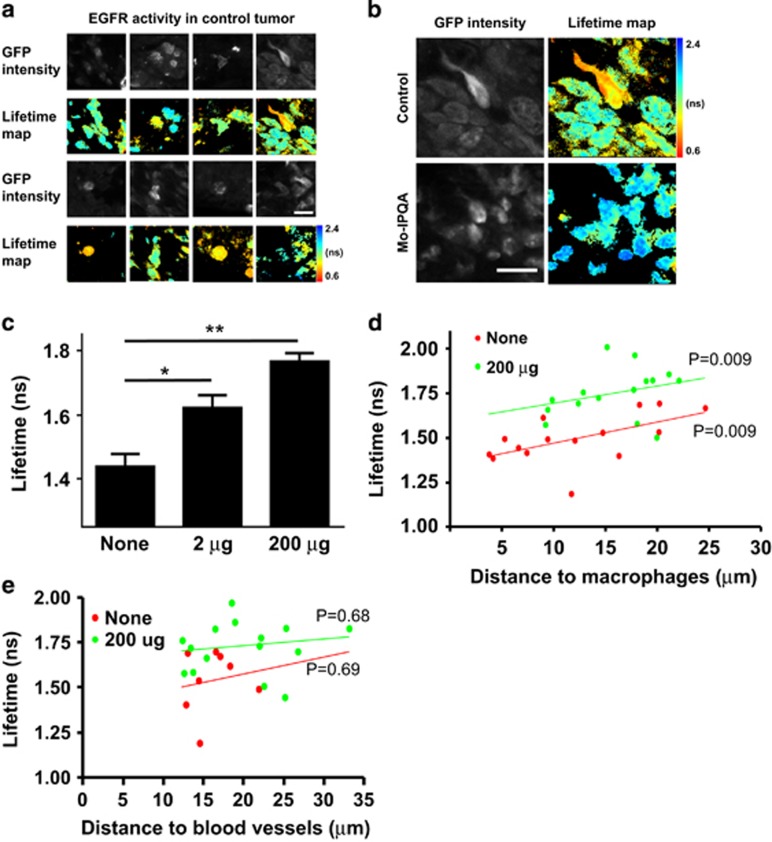
Effect of Mo-IPQA on EGFR activity in HCC1954 xenograft tumours monitored with Picchu-X biosensor. (**a**) FRET/FLIM images of Picchu-X biosensor expressed in untreated HCC1954 tumour cells (animal were injected with lipopolyplexes modified with **Y-I** peptide and peptide/DNA in a 2:1 ratio) and lifetime distribution among them. (**b**) Representative images of Picchu-X biosensor expressing cells from untreated and treated animals with Mo-IPQA (200-μg dose). (**c**) Quantification of lifetime changes in tumour cells after treatment of animals with Mo-IPQA at 2-μg and 200-μg doses. Data were expressed as means±s.e.m. (*P<0.005, **P<0.0001, Student’s *t*-test, tumours from four animals per group were imaged). (**d**) The association between proximity of the nearest macrophage to tumour cells and EGFR activity was evaluated by ANCOVA; in control and high-dose treated groups. (**e**) The association between proximity of the nearest CD31+ blood vessels to tumour cells and EGFR activity (*P* values presented for both ANCOVA models).

**Figure 6:**
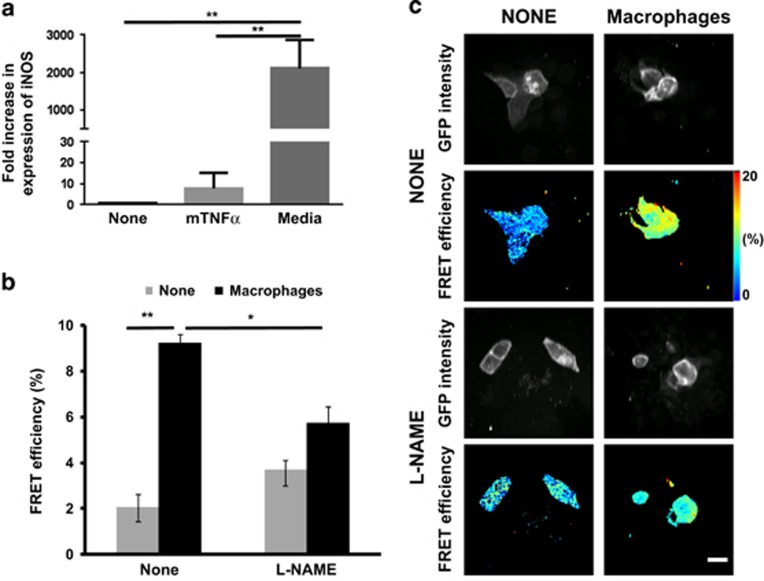
Effect of nitric oxide inhibitor on EGFR activity in HCC1954 cells co-cultured with activated macrophages. (**a**) mRNA level of *iNOS* was detected by qPCR (relative to the house keeping gene Tata-binding protein) in mouse macrophages treated with mouse tumour necrosis factor alpha (50 ng/ml) or conditioned media of HCC1954 cells for 3 days. One-way ANOVA to test differences between group means. *P* values are derived from a post-hoc Tukey’s test subsequently performed to compare means of individual groups while controlling for family-wise error rate. The difference was statistically significant between control and treatment groups, data were expressed as means±s.e.m. (*P<0.005, *N*=3). (**b**) HCC1954 cells expressing Picchu-X biosensor were co-cultured with activated macrophages from (**a**) for 48 h. Treated with L-NAME (2 mM) for 1 h, fixed and imaged. Presence of macrophages significantly increased FRET efficiency (**P<0.0001, *N*=6), which was significantly decreased by *iNOS* inhibitor (*P=0.01). (**c**) FRET/FLIM images of cells with and without L-NAME; and with and without activated macrophages. Scale bar is 25 μm.

We next assessed the levels of EGFR activity in untreated and Mo-IPQA-treated tumours *ex vivo*. We used 30-μm fixed sections of tumour for two-photon imaging to simultaneously capture the fluorescence lifetime of eGFP in tumour cells expressing the Picchu-X biosensor and to measure the distance of the biosensor-expressing tumour cells to the closest blood vessel and tumour-associated macrophage (TAM), which were stained with anti-CD31 and F4-80 antibodies, respectively. The fluorescence lifetime of eGFP in the absence of the mRFP1 acceptor fluorophore (Picchu-X-GFP control without mRFP1) in cells was 2.06±0.005 ns (*N*=30 images, from experiments presented in Figures 1 and 6) and 1.85±0.009 (*N*=18 images) in tissue (Supplementary Figure S11D). The fluorescence lifetime of eGFP (Picchu-X biosensor) in untreated tumours was 1.44±0.04 ns (Mean±s.e.m., *N*=15) suggesting active EGFR *in situ,* as observed by donor fluorescence lifetime quenching of the Picchu-X biosensor (Figure 5). Interestingly, the activity of EGFR was heterogeneous (Figure 5a) within the same untreated tumour section and did not depend on EGFR expression levels (Supplementary Figures S11A–B). Treatment with Mo-IPQA led to inhibition of EGFR activity in tumour cells (Supplementary Figure 5b), importantly in a manner dependent on the administered Mo-IPQA dose (Figure 5c). EGFR inhibition was demonstrated by a significant increase in the fluorescence lifetime of eGFP from 1.44±0.04 ns (untreated control) to 1.62±0.04 ns and to 1.77±0.02 ns, respectively, in mice treated with 2 μg Mo-IPQA and 200 μg Mo-IPQA.

The association between tumour cells and TAMs has been previously described.^31^ We analysed the association between proximity of TAM to tumour cells (*d*_*m*_) and EGFR activity, by assessing the correlation between *d*_*m*_ and Picchu-X biosensor lifetime, in the presence or absence of *in vivo* inhibition with Mo-IPQA. We performed ANCOVA with *d*_*m*_ and cell treatment as predictors of biosensor lifetime to statistically assess how the proximity of macrophage to tumour cells affects EGFR activity in the presence or absence of TKI treatment. We found that *d*_*m*_ remained strongly correlated with lifetime irrespective of cell treatment (*P*=0.009), suggesting that Mo-IPQA treatment did not modify this association. Similar regression slopes (control: 0.0113±0.0032 versus Mo-IPQA: 0.0097±0.0086) linking *d*_*m*_ to lifetime were observed between untreated control and the Mo-IPQA treated animals (Figure 5d, Supplementary Figure S11C). Similar statistical analyses did not show a relationship between EGFR lifetime in biosensor expressing tumour cells and distance to proximal blood vessels (*d*_*b,*_ Figure 5e, Supplementary Figure S11C) suggesting that unlike the proximity to TAMs, the distance of a given tumour cell to its nearest blood vessel does not significantly influence EGFR activity. In a colorectal cancer model (LIM1215 xenograft) we observed a significant reduction of tumour size following cetuximab treatment (from 0.16±0.03 cm^3^ in the control to 0.04±0.01 cm^3^ in the cetuximab treatment group, *P*=0.006). An alternative, antibody-based FRET assay for detection of protein phosphorylation in tissue^27^ was employed to assess EGFR activity in LIM1215 xenograft, due to a poor *in vivo* transfection efficiency. A significant cetuximab-associated reduction of FRET efficiency (Supplementary Figure S12B), comparing untreated control (FRET efficiency 4.1±0.006%) and cetuximab-treated tumour tissues (FRET efficiency 0.01±0.003%, *P*<0.0001), was found. We also show that EGFR activity measured by Picchu-X lifetime is consistent with EGFR activity measured by pEGFR-FLIM assay in cell-based experiment (Supplementary Figure S12A). Due to a high degree of infiltration by macrophages (in clusters) in LIM1215 xenograft, instead of distance to the nearest macrophage, we quantified the % of area occupied by macrophages in the field of view. We found a significant correlation between %mac and EGFR phosphorylation (Supplementary Figure S12C) in LIM1215 colorectal tumour xenograft, which further corroborates our finding in HCC1954 breast cancer model (Figure 5d). On the contrary, a non-small cell lung cancer cell line H1975-derived xenograft expressing mutant (L858R & T790M double mutation that renders EGFR constitutively active and resistant to erlotinib/gefitinib^32^) demonstrated constitutive EGFR activation that is independent of regulation by the tumour cell:macrophage proximity (Supplementary Figure S13).

It has been shown that nitric oxide can activate EGFR via s-nitrosylation.^33^ We found that pre-treatment of bone marrow-derived macrophages with conditioned media from HCC1954 cells led to a 2115±307% transcriptional upregulation of the *iNOS* mRNA in macrophages (Figure 6a). This is significantly more pronounced than the known *iNOS* gene transcriptional response triggered by inflammatory cytokines such as TNFα (Figure 6a). Co-culture of macrophages with HCC1954 cells resulted in significant activation of EGFR (Figures 6b and c). Addition of the *iNOS* inhibitor L-NAME significantly decreased the activation of EGFR in tumour cells co-cultured with macrophages (Figures 6b and c). These findings suggest that nitric oxide generation by activated macrophages can in part explain the dependence of EGFR activity on tumour cell-macrophage proximity *in vivo* (Figure 5d).

Taken together, our data indicate that the proximity of macrophages to tumour cells was positively associated with EGFR activity, and this association persists despite the inhibition of EGFR activity by a TKI.

## Discussion

This study demonstrates intra-tumoral heterogeneity of EGFR activity in a number of tumour models (breast, colorectal and lung), using an EGFR-targeted lipopolyplex to deliver DNA encoding for an EGFR biosensor, in conjunction with fluorescent lifetime imaging measurements. The EGFR-targeting peptide also mediates internalization of the lipopolyplex, thus enhancing transfection of the DNA and expression of the EGFR biosensor. An *ex vivo* approach gives proof of principle for the use of an EGFR biosensor to monitor the pharmacodynamic changes in EGFR activity in response to targeted therapy. This was coupled to the tumour tissue pharmacokinetic measurements of a radiolabelled TKI inhibitor. The mechanistic basis for the intratumoral heterogeneity of EGFR activity in this model of breast cancer was demonstrated to be related to the degree of macrophage infiltration in the tumour microenvironment.

Intravasation is the process whereby tumour cells invade into tumoral blood vessels and can be considered a rate-limiting step in the metastatic process. Tumour cells and macrophages are closely associated during intravasation^34^ and interact through a paracrine loop involving the macrophage growth factor CSF-1 released from tumour cells, and EGF released by macrophages.^35^ Measurement of the distance between biosensor-expressing tumour cells and macrophages in tumour sections showed a positive correlation between high EGFR activity and proximity to TAMs. When tumours were treated with Mo-IPQA, the correlation was maintained. The importance of the CSF-1/EGF paracrine loop in metastasis has been clearly demonstrated by mammary xenograft models with a conditional knockout of CSF-1.^35^

Our study demonstrates for the first time that the spatial relationship between tumour cells and macrophages, which is not uniform across the tumour bulk, contributes significantly towards the intratumoral, cell-to-cell heterogeneity of EGFR activity. Recently, there has been a substantial interest in the effect of TAMs on the efficacy of targeted therapies such as MAPK inhibitors and sorafenib and so on.^36, 37^ There are no data available, concerning the effect of pharmacological manipulation of macrophage function, on the therapeutic efficacy of EGFR TKI. Here we have found that tumour cell-conditioned media can upregulate *iNOS* mRNA transcription in bone marrow-derived macrophages. Co-culturing macrophages and basal-like breast cancer cells upregulated the EGFR activity within the tumour cells as measured by the Picchu-FLIM EGFR sensor. The macrophage-induced increase in tumour cell EGFR activity can be reduced significantly by an *iNOS* inhibitor, suggesting a novel mechanism of how proximity to macrophages in the microenvironment can regulate tumour cell signalling and hence give rise to the observed intratumor heterogeneity in EGFR signalling activity. Although the production of the nitric oxide-derived reactive nitrogen species peroxynitrite reactive nitrogen by macrophages has been shown previously to modify chemokines,^38, 39, 40^ the effect of such nitration effect on macrophage-induced EGFR activity has not been demonstrated before.

EGFR biosensing was first described by the Matsuda group more than a decade ago.^9^ However, the preclinical imaging of EGFR heterogeneity in animal models has been hampered by difficulties in targeting an EGFR biosensor to tumour cells *in vivo*. We have addressed this difficulty by using a unique liposome-based delivery system with an EGFR targeting peptide displayed at the surface of the lipopolyplex, achieving for the first time tumour cell-specific uptake and expression of the EGFR biosensor *in vivo*.

One of the potential problems with EGFR TKI and other targeted therapies can be the failure for the drug to reach its intended site. As tumour grows at an accelerated rate, interstitial fluid pressure increases. While drugs may flow through the vasculature surrounding the tumour, the combination of intact vasculature and high tumour interstitial fluid pressure can result in reduced or failed drug uptake.^41^ The importance of interstitial fluid pressure *in vivo* has been demonstrated by the use of platelet-derived growth factor receptor inhibitors that reduce intra-tumoral hypertension,^42, 43^ which lead to an improved uptake of the chemotherapeutic agent taxol. Using [^125^I]-Mo-IPQA we have shown, by the corresponding reduction on the fluorescence lifetime of the expressed EGFR biosensor in all regions of interest when higher doses of TKI were used, that drug delivery is dose-dependent. Protein activity biosensing and tumour tissue pharmacokinetic measurements, afforded by quantifying the tumour uptake of a radiolabelled inhibitor, can therefore be coupled to interstitial fluid pressure measurements to fully understand the heterogeneity of treatment response among different tumour loci within the same animal as we recently reported using sodium iodide symporter SPECT imaging.^44^ In other cancer types such as metastatic colorectal tumours, only 10–20% of patients with wild type *KRas/BRAF* respond to anti-EGFR monotherapy using monoclonal antibody therapies (for example Cetuximab against EGFR^45^). Factors besides tissue pharmacokinetics, such as between-patient heterogeneity in FcγR-dependent innate immune function as well as target receptor availability (which may be independent of the RTK activity), are likely to also have an important influence on the efficacy of EGFR antibody treatment.^46^

The heterogeneity of signalling activity between individual cell populations within a tumour, which persists in the presence of treatment (in this case an EGFR TKI, Figure 5d), may culminate in a potentially treatment-resistant cell subpopulation and may therefore provide an explanation for therapeutic failures.^47^ Further investigation of the observed macrophage proximity-dependent heterogeneity of EGFR activity between tumour cell subpopulations, will therefore guide the development of future strategies that may combine molecule-targeted cancer therapy with immunomodulatory agents (for example against TAM, which may have been shown to have tumour promoting function in breast and other tumour types^48^).

## Materials and methods

### Tissue culture, reagents and antibodies

Basal-like breast cancer cell-lines, MDA-MB-231 (purchased from ATCC) and HCC1954 (generous gift of Dr Andrew Tutt, King’s College London, UK), independently validated by STR DNA fingerprinting at The Institute of Cancer Research (London, UK), were maintained in DMEM and RPMI-1640, respectively (Life Technologies Ltd, Paisley, Uk) and tested to be mycoplasma free. Bone marrow macrophages were extracted from HCC1954 tumour bearing mice, cultured with macrophage colony stimulating factor-1 (GFM8-100, Cambridge Bioscience, UK) for 7 days before use in experiments. Antibodies: anti-EGFR (clone D38B1), anti-p-Y1173-EGFR (clone 53A5), anti-p-Y221-CRKII (#3491) and CRKII (#3492) were from Cell Signalling (Danvers, MA, USA); anti-CD31 (clone 2H8) from AbD Serotec Ltd (Oxford, UK); Anti-F4/80 (clone A3-1) from Abcam (Cambridge, UK). Goat anti-rat-Alexa647 (415-605-166-JIR) and goat anti-hamster-Cy3 (307-165-003-JIR) were from Stratech Scientific, UK. 1,2-Dioleoyl-sn-glycero-3-phosphoethanolamine (DOPE) was from Avanti Polar Lipids Inc. (Alabaster, AL, USA) and Cy5-NHS was from GE Healthcare (Amersham, UK). Cholesterol and all general reagents for chemical synthesis were from Sigma-Aldrich (Dorset, UK). *N*-(2-(2-(2-(2-hydroxyethoxy)ethoxy)ethoxy)ethyl)-*N,N*-dimethyl-2,3-bis((*Z*)-octadec-9-enyloxy)propan-1-aminium (DODEG4)^49^ and *N,N,N*-trimethyl-2,3-bis((*Z*)-octadec-9-enyloxy)propan-1-aminium (DOTMA)^50, 51^ were synthesized as previously described. EGFR TKI PD168393 was from Santa Cruz Biotech (Heidelberg, Germany).

General methods for chemical synthesis are included in the Supporting Information. The EGFR inhibitor (*E*)-but-2-enedioic acid [4-(3-iodoanilino)-quinazolin-6-yl]-amide-(3-morpholin-4-yl-propyl)-amide (Mo-IPQA) was synthesized^28^ and its HPLC and ^1^H NMR characterization data were identical to the literature (Supplementary Supporting Figure S0). Synthesis of morpholino-[^125^I]-IPQA was carried out as previously described.^28, 52^ Cy-5 DOTMA was synthesized from a previously described intermediate^51^ in two steps (Supplementary Supporting Information). The four bifunctional peptides used in this study (Figure 2) were synthesized using standard solid-phase peptide synthesis techniques and purified by HPLC. Purity and identity of the peptides were assessed by analytical HPLC and electrospray mass spectrometry (Supplementary Supporting Figures S1–S8).

### Preparation and characterization of liposomes

All lipid components were dissolved in CHCl_3_ to a concentration of 1 mM. The lipid quantities were mixed together in the following molar ratios: DODEG4 39 mol% DOPE 23 mol% DOTMA 15 mol% cholesterol 23 mol%. The solvent was then removed under reduced pressure to form a thin film, which was further dried on a high vacuum line for 2 h. This was hydrated with sterilized water to the desired total lipid concentration, sonicated for 10 min, and used immediately. Methods for the characterization of the liposomes are described in the Supplementary Supporting Information, and the DLS and zeta potentials are given in Supplementary Table S1.

Lipopolyplexes used for *in vitro* experiments were formulated as described (including Cy5-DOTMA) at a total lipid-cholesterol concentration of 130 μM using a bath sonicator. For every 100 μL of sonicated liposome solution, 4–16 μg of peptide was added followed by 1–4 μg of plasmid DNA coding for the Picchu-X sensor. The samples were mixed and used immediately.

The lipopolyplexes used for the *in vivo* studies were prepared as described (without the inclusion of the Cy5-DOTMA) at a total lipid concentration of 1.95 mM using a probe sonicator (Sonifier SLPe, Branson, USA; 10 min continuous pulse). Two equivalents of peptide **Y-I** (1–5 mM solution made from stock in distilled water—w/w relative to the DNA sensor) were added to the liposome solution (usually 136 μg per mouse to give a 2:1 ratio). This solution was then diluted to an overall lipid concentration of 0.5 mM using sterilized water. The desired amount of DNA (68 μg per mouse) was then separately diluted to a concentration of 0.2 μg/μl and added to the liposome solution. The sample was allowed to sit for 10 min after gentle mixing. The sample was then concentrated to an appropriate volume (1.95 mM total lipid concentration) to allow injection into the mouse models (100 μl per mouse) using VivaSpin columns (Molecular Weight Cut Off 10 000, GE Healthcare Life Science Ltd, Amersham, UK).

### *In vitro* liposomal transfection

13 μM liposome solution, containing 0.4 μg of sensor plasmid DNA and 1.6 μg of peptide per 100 μl, was applied to cells at 60% confluence in six-well-plates. Following 4 h incubation at 37 °C, cells were washed twice with sterile PBS to remove unbound liposomes and incubated with normal growth medium for 48 h to allow protein expression. All *in vitro* experiments were repeated at least three times.

### *In vivo* experimentation

Subcutaneous xenograft animal models of cancer (H1954 cells for breast, H1975 cells for lung and LIM1215 cells for colorectal in Charles River female CD1-Foxn1 nu/nu mice (6–8 weeks old)) were used in this study. The objective was to investigate the effect of EGFR-specific TKI on EGFR activity *in situ* using an intravenously administered liposome-encapsulated EGFR biosensor. All animal studies were approved by the University College London and King’s College London Biological Services Ethical Review Committee and licensed under the UK Home Office regulations (PPL no: 70/7350) and the Animals (Scientific Procedures) Act 1986 (Home Office, London, United Kingdom), informed by the National Cancer Research Institute Guidelines.^53^ Animal weights and tumour volumes were recorded every 2 days. Cohort sizes of at least four mice per treatment group were used (according to previously published method^54^). All mice used for experiments were female and randomly assigned to non-blinded treatment groups. Experiments were performed in at least duplicate. Treatment with clinical-grade cetuximab (Erbitux, Merck, Feltham, UK) was 20 mg/kg IP biweekly for 2 weeks.

### Single-photon emission computed tomography (SPECT)/CT imaging of Mo–IPQA clearance in control mice

Two mice (6–8 weeks old) were anaesthetized with an isoflurane/O_2_ mix and a tail vein cannulated for the delivery of approximately 7 MBq of ^125^I-Mo-IPQA (equates to 28 ng) in 300 μl sterile saline.

### Tumour induction, *in vivo* lipopolyplex transfection, whole body Mo-IPQA biodistribution and tumour uptake

Mice were injected subcutaneously on the right flank with 3 × 10^6^ HCC1954 cells in 100 μl serum-free media. Tumours were measured using a calliper. Tumour volumes were estimated assuming an ellipsoid shape using the formula volume=length × width × depth × π/6. Once tumours reached approximately 200 mm^3^, 100 μl of a1.95 μM lipopolyplex solution containing 136 μg of peptide and 68 μg Picchu-X (or Picchu-X-GFP control) DNA were injected i.v. into the tail vein. After 48 h mice received i/v injection of saline with 2 μg Mo-IPQA or 200 μg Mo-IPQA. Both the 2 μg and 200 μg doses contained 2 ng of ^125^I-Mo-IPQA with a dose of 0.5 MBq/mouse. After 1 h, the mice were exsanguinated by cardiac puncture and culled via a schedule 1 method. Various organs were harvested from each animal, weighed and gamma counted (PerkinElmer, Coventry, UK). The tumours were then frozen in OCT for histology.

### Microscopy

Confocal fluorescence images were acquired on a confocal fluorescence laser-scanning microscope (model LSM 510; Carl Zeiss Inc., Cambridge, UK) equipped with a × 63/1.4NA Plan-APOCHROMAT oil immersion objective. Fluorescence Lifetime images of Picchu-X-transfected cells in cell culture or tissue sections from mouse xenografts were captured on a multiphoton microscope, and analysed as previously described.^55, 56^

### Statistical analyses

Student’s *t*-test was employed to assess changes in biosensor lifetime following treatment with Mo-IPQA. The distances between biosensor-expressing tumour cells and nearby macrophage (*dm*) and as well as blood vessels (*db*) biosensor-expressing tumour cells (d) were assessed in relation to biosensor lifetime by simple linear regression in all imaged cells (*n*=29). For any significant association, analysis was repeated, in each cell group separately (control, Mo-IPQA-treated). Regression coefficients or slopes in the control and Mo-IPQA-treated group were further compared through a test for interaction by incorporating a product of *dm* or *db* and cell treatment in the regression model. The association between *dm* or *db*, cell treatment and lifetime was further assessed using analysis of covariance (ANCOVA), with *dm* or *db* and cell treatment as predictors of lifetime. Statistical significance was set at *P*<0.05. The above analyses were performed with R version 3.1.1 (R Foundation for Statistical Computing).
