# Supplementary material for: Detecting intratumoral heterogeneity of EGFR activity by liposome-based in vivo transfection of a fluorescent biosensor
Source: Oncogene. 2017 Feb 6;36(25):3618–28. doi: 10.1038/onc.2016.522 (PMC5421598; doi:10.1038/onc.2016.522)
Supplement: Supplementary Materials [file onc2016522x1.docx]

**Detecting intratumoral heterogeneity of EGFR activity by liposome-based *in vivo* transfection of a fluorescent biosensor**

Gregory Weitsman*, Nicholas J. Mitchell*, Rachel Evans, Anthony Cheung, Tammy L. Kalber, Robin Bofinger, Gilbert O. Fruhwirth, Melanie Keppler, Zoë V.F. Wright, Paul R. Barber, Peter Gordon, Tamara de-Koning, Wahyu Wulaningsih, Kerstin Sander_,_ Borivoj Vojnovic, Simon Ameer-Beg, Mark Lythgoe, James Arnold, Erik Årstad, Helen C. Hailes, Alethea B. Tabor and Tony Ng

**Supporting Information**

**Table of Contents**

| General methods | 2 |
| --- | --- |
| Synthesis of Cy5-DOTMA | 3 |
| Radiosynthesis of morpholino-[^125^I]IPQA | 5 |
| Figure S0 Analytical HPLC and NMR of Mo-IPQA | 8 |
| Solid phase peptide synthesis | 9 |
| Analytical data for peptides (Figures S1-S8) | 11 |
| Characterisation of liposomes | 15 |
| Table S1: DLS and zeta potentials of lipoplexes  Design and construction of Picchu-X.  qRT-PCR and primers.  Single-photon emission computed tomography (SPECT)/CT imaging (instrumentation setting) | 16  16  16  17 |
| Figure S9. Picchu-X phosphorylation in response to EGF stimulation. | 18 |
| Figure S10. Effect of different targeting peptides on transfection | 19 |
| Figure S11. Effect of EGFR expression on Picchu-X signal in tumour  Figure S12. EGFR activity imaging by Picchu-FLIM in LIM1215 cells and antibody based pEGFR FLIM assay in xenografts.  Figure S13. EGFR activity imaging by Picchu-FLIM in H1975 xenografts. | 20  21  23 |
| References | 24 |

**General Methods:** Reagents for chemical synthesis were purchased from Sigma-Aldrich Co. Ltd. unless otherwise statedand used without further purification. All reagents were of commercial quality and used as received and all solvents anhydrous. Cy5-NHS was purchased from GE Healthcare (Amersham, UK) and NaI [^125^I] was generated at Perkin Elmer. Thin Layer Chromatography (TLC) was performed on aluminium backed Sigma-Aldrich TLC plates with F254 fluorescent indicator. Visualisation was performedby quenching of UV fluorescence or by staining the plates with potassium permanganate solution (1.5 g KMnO_4_,10 gK_2_CO_3_, 1.25 mL 10% NaOH in200 mL water). Normal phase flash chromatography was carried out using silica gel (43–60 μm) supplied by Merck. Preparative and analytical HPLC was performed on a Varian ProStar HPLC system with a Model 210 solvent delivery module and a Model 320 UV detector. The analysis of the chromatograms was conducted using Star Chromatography Workstation software Version 1.9.3.2. Preparative purificationwas performed using a Varian column (21.2 x 100 mm, C18, 5 μm beads, flow rate of 10 mLmin^-1^) or a Gemini column (4.6 x 250 mm, C18, 5 μm beads, flow rate of 4 mLmin^-1^). Analytical HPLC was performed usinga Varian column (2.1 x 250 mm, C18, 5 μm beads, flow rate of 1 mL min^-1^). Asolvent system of water (0.1% TFA) as solvent A and acetonitrile (0.1% TFA) as solvent B was used with a gradient of 2– 98% B over 20 minunless otherwise stated.The radiolabelled compound Morpholino-[^125^I]IPQAwas purified usinga Zorbax column (300SB-C18, 9.4 x 250 mm, 5 µm) using a solvent system of water (0.1% TFA) as solvent A and methanol (0.1% TFA) as solvent B, using a gradient of 2 – 40% B over20 minwith a flow rate of 3 mLmin^-1^ unless otherwise stated.ESI-MS analysis was performed on a Waters Acquity Ultra Performance LC/MS system. MALDI MS was performed on a Waters MALDI MicroMX instrument using α-cyano-4-hydroxycinnamic acid (CHCA) or sinapinic acid (SA) as the matrix (1 mg/mL in methanol). NMR (^1^H and ^13^C) was performed on a 600 MHz AMX Bruker Spectrometer. The chemical shifts (δ) were given in units of ppm relative to tetramethylsilane (TMS), where δ (TMS) = 0 ppm. Coupling constants (*J*) were measured in Hertz (Hz), multiplicities for ^1^H coupling are shown as s (singlet), d (doublet), t (triplet), m (multiplet), or a combination of the above. Deuterated chloroform (CDCl_3_), dimethylsulfoxide (d_6_-DMSO) and methanol (CD_3_OD) were used as solvents (as stated) for all NMR analysis.

**Synthesis of Cy-5 DOTMA**

***N*-(3-aminopropyl)-*N,N*-dimethyl-2,3-bis((*Z*)-octadec-9-enyloxy)propan-1-aminium bromide**


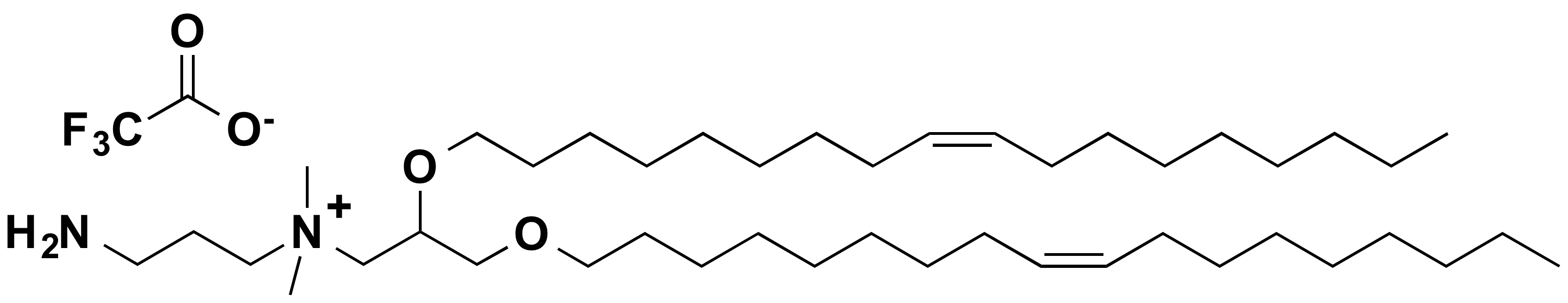


To *N,N*-dimethyl-2,3-bis((*Z*)-octadec-9-enyloxy)propan-1-amine (1.35 g, 2.18 mmol) ([1](#_ENREF_1)) in anhydrous acetone (10 mL) was added *tert*-butyl 3-bromopropyl-carbamate(1.04 g, 4.36 mmol) ([2](#_ENREF_2)) also in anhydrous acetone (10 mL) and the solution was heated in a sealed tube at 100 °C for 48 h. After this time the solvent was removed under reduced pressure and the crude material dissolved in a 1:1 solution of TFA:CH_2_Cl_2_(10 mL) and stirred at room temperature for 3 h.The solvent was again removed and the crude product was dissolved in sat. NaHCO_3_ (20 mL) and extracted into CH_2_Cl_2_ (20 mL), dried (Na_2_SO_4_) and concentrated. The material was purified *via* flash column chromatography (5 – 20 % methanol in CH_2_Cl_2_) to afford an oil which was dried under high vacuum to give the desired product (1.15 g, 67%). R_f_ 0.39 (20% MeOH in CH_2_Cl_2_); ν_max_; (CHCl_3_)/cm^-1^ 2923, 2854, 1678; ^1^H NMR (600 MHz, CDCl_3_)δ/ppm 0.86 (t, 6H, *J*= 7.1 Hz, CH_2_*CH_3_*), 1.24-1.31 (m, 44H, C*H_2_*), 1.53 (m, 4H, C*H_2_*CH_2_O), 1.82-2.00 (m, 10H, C*H_2_*CH=CHC*H_2_* and NCH_2_C*H_2_*CH_2_NH_2_), 3.36-3.72 (m, 18H, C*H_2_*OC*H_2_*CHO*CH_2_*, C*H_2_*N(*CH_3_*)_2_, and N*CH_2_*CH_2_*CH*_2_NH_2_), 4.01 (brm, 1H, OCH_2_C*H*CH_2_N(CH_3_)_2_), 5.30-5.36 (m, 4H, CH_2_C*H=*C*H*CH_2_); ^13^C NMR (150 MHz, CDCl_3_) δ/ppm 14.25, 20.94, 22.81, 25.13, 26.07, 26.12, 26.24, 27.35, 27.74, 29.21-30.07 (signals superimposed), 32.01, 32.76, 33.82, 36.75, 39.33, 50.99, 51.64, 53.20, 59.27, 63.19, 66.37, 68.63, 69.47, 72.13, 73.12, 115.77, 117.71, 129.91-130.48 (signals superimposed), 161.92, 162.15; *m/z*  (ESI-HRMS) found 677.6918, C_44_H_89_N_2_O_2_ [M+H]^+^ requires 677.6924.

***N*-(3-aminopropyl)-*N,N*-dimethyl-2,3-bis((*Z*)-octadec-9-enyloxy)propan-1-aminium - Cyanine 5**


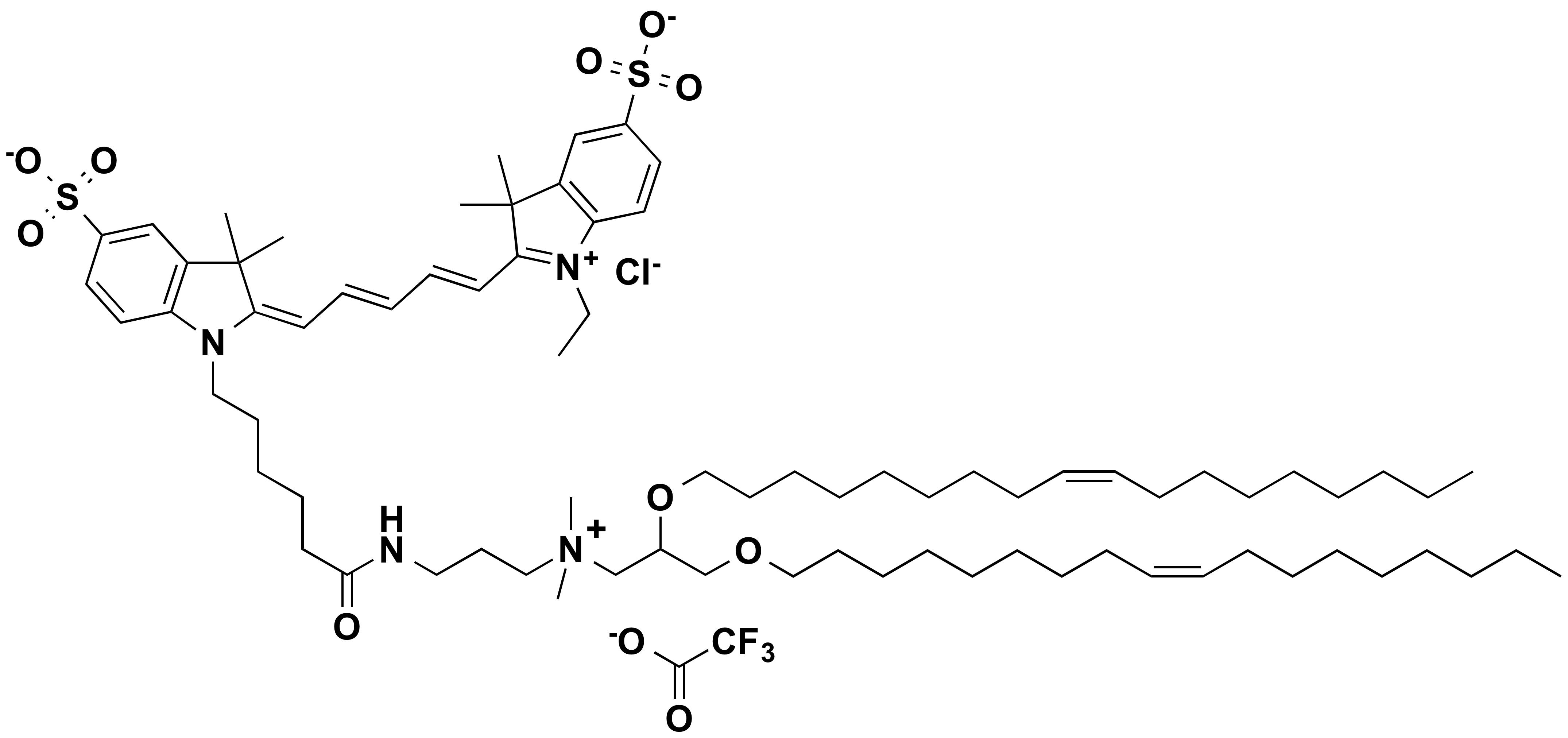


To Cy5-NHS (1 mg, 1.26 mmol) in anhydrous CH_2_Cl_2_(2 mL) was added triethylamine (0.2 mL, 1.52 mmol) followed by the starting lipid (1.2 mg, 1.52 mmol), in CH_2_Cl_2_ (2 mL). The reaction was stirred at room temperature for 18 h in the dark. The solvent was removed under reduced pressure and the crude product purified *via* preparative TLC(20% methanol in CH_2_Cl_2_)to afford the desired product (1.8 mg, 81%). R_f_ = 0.63 (20% methanol in CH_2_Cl_2_); *m/z* (MALDI: matrix sinapinic acid (1 mg/mL in methanol)) found 1315.0, C_77_H_126_N_4_O_9_S_2_[M+H]^+^requires 1314.9.

**Radiosynthesis of [^125^I]-morpholino-IPQA**

***N*^4^-(3-(trimethylstannyl)phenyl)quinazoline-4,6-diamine (**[**3**](#_ENREF_3)**)**

The reaction was carried out under anhydrous conditions. To*N*^4^-(3-iodophenyl)quinazoline-4,6-diamine (100 mg, 0.276 mmol) was added Pd(PPh_3_)_4_ (32 mg, 27.6 μmol), anhydrous 1,4-dioxane (10 mL) followed by (SnMe_3_)_2_(181 mg, 0.55 mmol)in anhydrous dioxane (114 μL) . The solution was heated to reflux, a further portion of Pd(Ph_3_)_4_ (32 mg, 10 mol%) added and the reaction allowed to reflux for 18 h. LCMS analysis showed the complete conversion of the starting material to the product. The solvent was removed under reduced pressure and the crude product re-dissolved in CH_2_Cl_2_ (5 mL) washed with water (3 x 5 mL), brine (3 x 5 mL) and dried (MgSO_4_). The product was purified *via* flash column chromatography (2% methanol inCH_2_Cl_2_)to afford the desired product (55 mg, 51%). ^1^H NMR (600 MHz, CDCl_3_)δ/ppm0.33 (m, 9H), 4.05 (m, 2H), 6.97 (d,*J* = 1.8 Hz, 1H),7.17 (br s, 1H), 7.22 (dd, *J* = 8.8, 1.8 Hz, 1H), 7.28 (m, 1H), 7.42 (t, *J* = 7.0Hz, 1H), 7.65 (s, 1H), 7.74 (d, *J* = 7.0 Hz, 1H), 7.83 (d, *J* = 7.4 Hz, 1H), 8.60 (s, 1H);^13^C NMR (150 MHz, CDCl_3_) δ/ppm -9.3, 31.1, 31.4, 53.6, 10.1, 116.3, 122.0, 123.9, 128.6, 128.7, 130.4, 131.9, 138.2, 143.4, 144.3, 145.3, 151.9, 156.2; *m/z* (ESI^+^)found 400.79, C_17_H_20_N_4_Sn [M+H]^+^ requires 400.09.

**But-2-enedioic acid (3-morpholin-4-propyl)-amide[4-(3-tributylstannyl-phenyl-amino)-quinazolin-6-yl]-amide**

To 3-(3-morpholin-4-yl-propylcarbamoyl)-acrylic acid ([3](#_ENREF_3)) (34 mg 0.1 mmol, as the triethylamine salt) in anhydrous pyridine (1 mL) was added EDC.HCl (23 mg, 0.12 mmol in 1 mL pyridine) and *N*^4^-(3-tributylstannylphenyl)-quinazoline-4,6-diamine (40 mg, 0.1 mmol, in 1 mL pyridine). The solution was stirred for 2 h at room temperature. A further portion of EDC.HCl (10 mg, 0.5 eq) and 3-(3-morpholin-4-yl-propylcarbamoyl)-acrylic acid (17 mg, 0.5 eq) were then added and the solution stirred at room temperature for 18 h. The solvent was removed under reduced pressure and the crude purified *via* silica flash chromatography (2 – 5% methanol in CH_2_Cl_2_).The product (42 mg) was isolated and further purified *via* HPLC (2 – 40% B over 30 min). The pure compound was re-dissolved in 5 mL of HPLC grade water and lyophilised to afford the desired product as a yellow solid (40 mg, 16%). ^1^H NMR (600 MHz, CDCl_3_) δ/ppm 0.31 (m, 9H), 1.80 (quin. *J* = 7.1 Hz, 2H), 2.49 (m, 6H), 3.37 (m, 2H), 3.71 (m, 4H), 7.10 (d, *J* = 15.0 Hz, 1H), 7.14 (d, *J* = 15.0 Hz, 1H),7.30 (d, *J* = 6.0 Hz, 1H), 7.38 (m, 2H), 7.75 (m, 2H), 7.83 (m, 1H), 8.44 (s, 1H), 8.77 (d, *J* = 2.0 Hz, 1H); ^13^C NMR (150 MHz, CDCl_3_) δ/ppm 9.9, 9.2, 27.1, 38.9, 54.6, 57.4, 67.4, 113.1, 116.9, 117.1, 119.0, 124.3, 124.5, 125.9, 128.1, 128.9, 129.4, 129.8, 129.9, 131.2, 133.3, 133.9, 135.4, 137.9, 139.5, 144.0, 147.6, 154.9, 159.8, 159.8, 164.8, 166.4;*m/z* (ESI^+^) found 624.0, C_28_H_36_N_6_O_3_Sn [M+H]^+^ requires 624.35.

**[^125^I]-morpholino-IPQA**

To a solution of [^125^I]NaI (approx. 2.4 MBq) in water (100 μL) was added HCl (250 μL, 0.1 M) followed by but-2-enedioic acid (3-morpholin-4-propyl)-amide[4-(3-tributylstannyl-phenyl-amino)-quinazolin-6-yl]-amide (1 mg/mL in ethanol, 200 μL) and H_2_O_2_ (50 μL at 3%). The solution was allowed to sit at room temperature for 30 min before the addition of 400 μL water. The analytical radiochemical yield as analysed by radio-HPLC was found to be 75%. For *in vivo* studies, the tracer was isolated and reformulated in sterile saline containing less than 5% ethanol. Morpholino-[^125^I]IPQA was obtained in 26% overall yield, and with a specific activity of 15.7 GBq/µmol.

**Morpholino_IPQA**

Mo-IPQA was synthesized according to literature procedures ([3](#_ENREF_3)). The purity of the compound was verified *via* RP-HPLC and ^1^H NMR; the acquired NMR spectra was consistent with the published data.

**
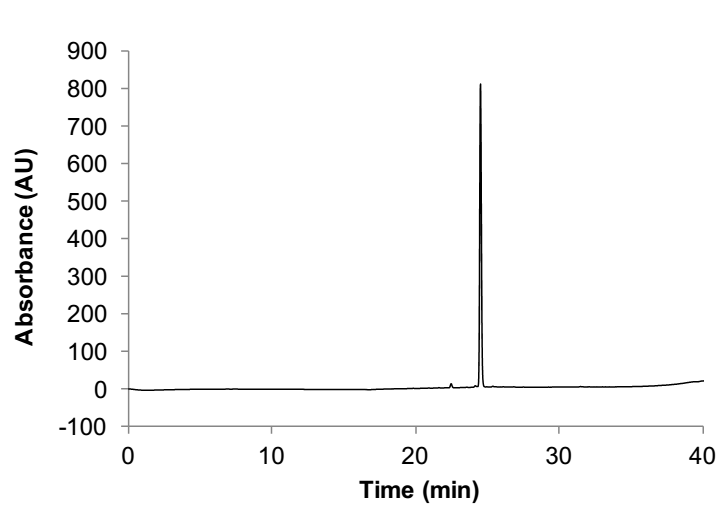
**

**
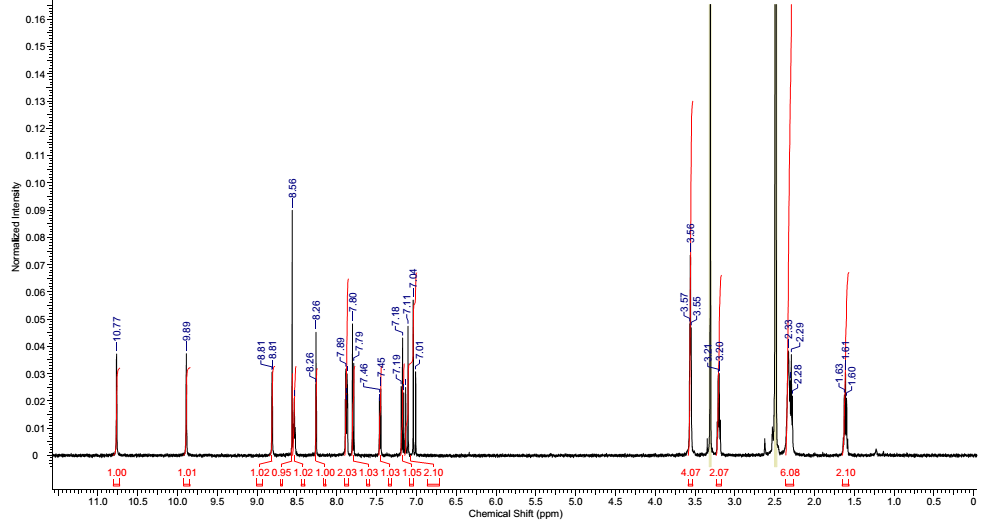
**

DMSO

Water

**Figure S0.**a) Analytical HPLC trace of purified Morpholino-IQPA; R_t_24 min, (20-40% B over 30 min, λ = 214 nm) b) ^1^H NMR of Morpholino-IPQA.

**Solid Phase Peptide synthesis**

**General methods.** All peptides were synthesized using standard Fmoc solid-phase peptide synthesis on a MutiSynTech Syro I automated system. Pre-loaded Wang resin was used with standard HBTU/DIPEA coupling chemistry (detailed below). All resins were pre-swelled in DMF for 30 min prior to the start of the synthesis. The total volume of all reagents in each step was 1.5 mL. *N*-Fmoc-protected amino acids were purchased from Novobiochem. Where required, the following side-chain protected amino acids were used: Fmoc-Lys(Boc)-OH; Fmoc-Arg(Pbf)-OH; Fmoc-Tyr(tBu)-OH; Fmoc-His(Trt)-OH; Fmoc-Thr(tBu)-OH.

*Fmoc deprotection:* Each deprotection step was allowed to proceed for 3 min with agitation for 20 s every minute in 40% piperidine in DMF at room temperature. The reagents were removed by filtration under vacuum and the resin washed with DMF (4 x 1.5 mL). The deprotection step was repeated using 20% piperidine in DMF with agitation for 20 s every minute for 10 min. The reagents were removed by filtration under vacuum and the resin washed with DMF (6 x 1.5 mL).

*Amino acid coupling:* To the resin was added 4 eq of the amino acid, 4 eq of HBTU and 8 eq of DIPEA to a total volume of 1.5 mL. The mixture was agitated for 20 s every 3 min for a total of 40 min at room temperature. The reagents were removed by filtration and the resin washed with DMF (4 x 1.5 mL).

*Peptide Cleavage:* After completion of the synthesis the peptide sequence was deprotected and cleaved from the resin by incubation in TFA/TES/EDT/H_2_O (2500:150:75:150 μL) for 3 h at room temperature. The cleavage cocktail was drained into a 15 mL falcon tube, the collected solution was mixed with diethyl ether and the peptide allowed to precipitate at -20°C for 10 min. The suspension was centrifuged at 4000 rpm for 5 min, the supernatant was discarded and the pellet re-dissolved in diethyl ether. This purification process was repeated three times after which the precipitate was dissolved in 0.1% TFA containing HPLC grade water, frozen in liquid N_2_ and freeze-dried overnight. The peptides were purified using the HPLC system and columns specified in the general methods section. Fractions containing the correct peak were pooled, the solvent removed under reduced pressure to < 5 mL, and the solution freeze-dried overnight. The resulting crystalline powder was analyzed *via* HPLC and ESI-MS.

**K_16_RVRRYHWYGYTPQNVI (Y-I)**


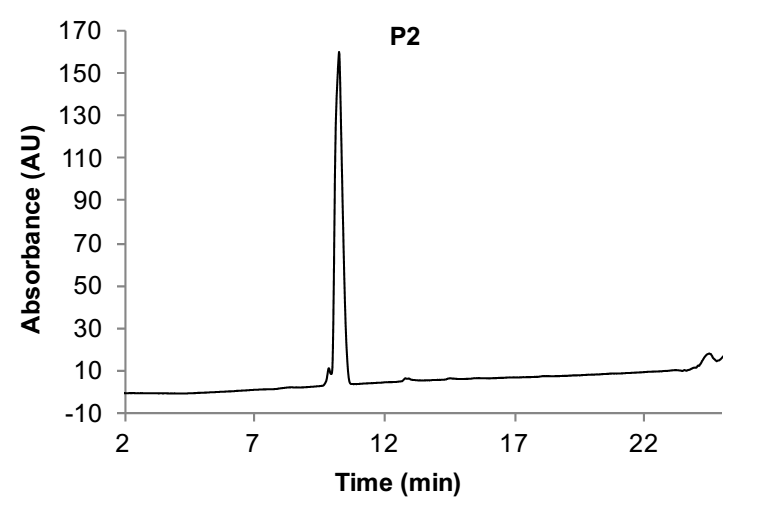


**Figure S1.** Analytical HPLC trace of purified K_16_RVRRYHWYGYTPQNVI; R_t_10.22 min,(2-60% B over 20 min, λ = 214 nm).

**Figure S2.***m/z*- Calculated Mass (ESI^+^);1040.15 [M+4H]^4+^,832.32 [M+5H]^5+^,693.77 [M+6H]^6+^,594.80 [M+7H]^7+^,520.58 [M+8H]^8+^,462.85 [M+9H]^9+^; Mass Found (ESI^+^); 1040.7 [M+4H]^4+^, 832.5 [M+5H]^5+^, 694.0 [M+6H]^6+^, 595.0 [M+7H]^7+^,520.6 [M+8H]^8+^, 462.9 [M+9H]^9+^.

**K_16_RVRRLARLLT (L-T)**


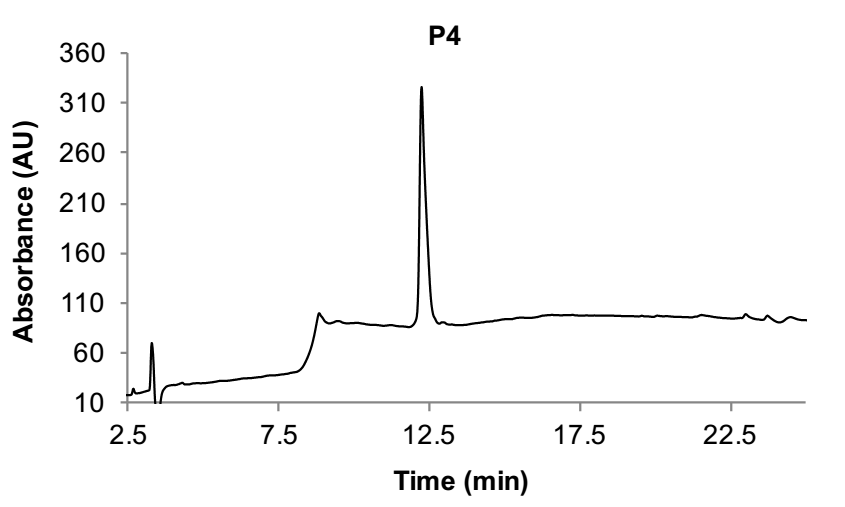


**Figure S3.** Analytical HPLC trace of purified K_16_RVRRLARLLT; R_t_ 12.22 min (2-30% B over 20 min, λ = 214 nm).

**Figure S4.***m/z*- Calculated Mass (ESI^+^); 1101.78 [M+3H]^3+^,826.59 [M+4H]^4+^, 661.47 [M+5H]^5+^, 551.39 [M+6H]^6+^, 472.76 [M+7H]^7+^, 413.79 [M+8H]^8+^;Mass Found (ESI^+^); 1102.05 [M+3H]^3+^,826.73 [M+4H]^4+^, 661.60 [M+5H]^5+^, 551.48 [M+6H]^6+^, 472.75 [M+7H]^7+^, 413.85 [M+8H]^8+^.

**YHWYGYTPQNVIRVRRK_16_ (I-Y)**


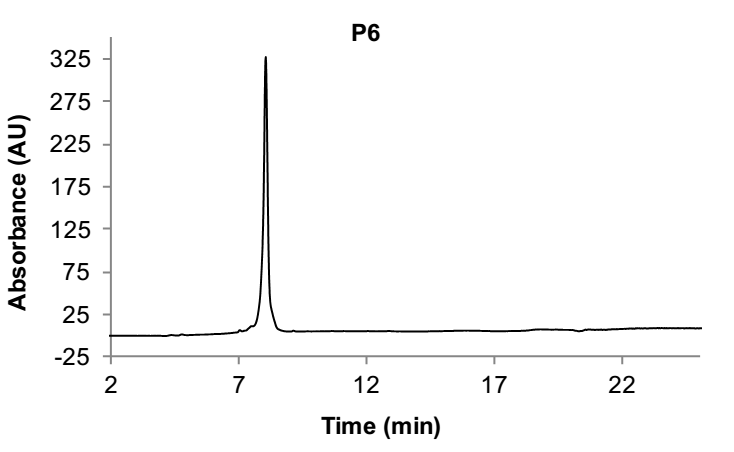


**Figure S5.** Analytical HPLC trace of purified YHWYGYTPQNVIRVRRK_16_; R_t_8.05 min (2-98% B over 20 min, λ = 214 nm).

**Figure S6.***m/z* - Calculated Mass (ESI^+^); 1040.15 [M+4H]^4+^, 832.32 [M+5H]^5+^, 693.77 [M+6H]^6+^, 594.80 [M+7H]^7+^, 520.58 [M+8H]^8+^, 462.85 [M+9H]^9+^; Mass Found (ESI^+^); 1040.35 [M+4H]^4+^, 832.47 [M+5H]^5+^, 693.95 [M+6H]^6+^, 594.96 [M+7H]^7+^, 520.59 [M+8H]^8+^, 462.83 [M+9H]^9+^.

**LARLLTRVRRK_16_(T-L)**

_
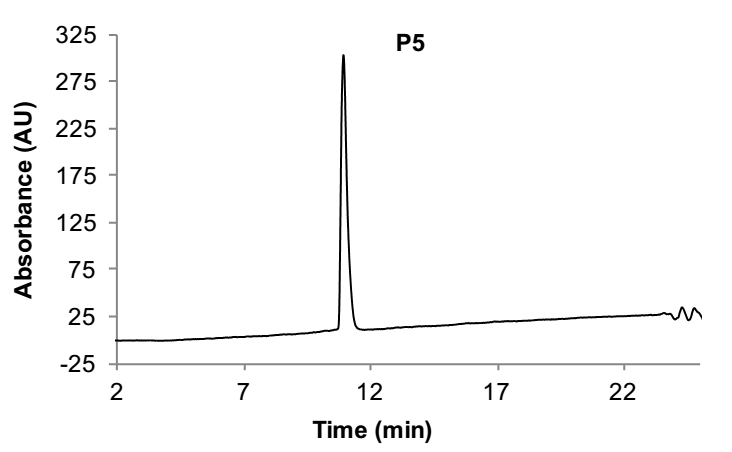
_

**Figure S7.** Analytical HPLC trace of purified LARLLTRVRRK_16_; R_t_10.88 min (2-50% B over 20 min, λ = 214 nm).

__

**Figure S8.***m/z* - Calculated Mass (ESI^+^); 1101.78 [M+3H]^3+^, 826.59 [M+4H]^4+^, 661.47 [M+5H]^5+^,551.39 [M+6H]^6+^, 472.76 [M+7H]^7+^, 413.79 [M+8H]^8+^; Mass Found (ESI^+^); 1102.03 [M+3H]^3+^,826.78 [M+4H]^4+^, 661.68 [M+5H]^5+^, 551.56 [M+6H]^6+^, 472.67 [M+7H]^7+^.

**Characterisation of liposomes**

The liposomes were characterized using dynamic light scattering (DLS) and zeta potential measurements. Data were obtained using a Malvern Zetasizer Nano-ZS (Malvern, UK); aliquots of 25 – 50 µL were diluted to 1 mL in sterilized water and analysed in triplicate at 25 °C. For the *in vivo* experiments the lipopolyplexes were concentrated in order to provide a volume appropriate for injection, and the size and surface charge were characterised to ensure reproducibility. The LPD particles must be < 150 nm in diameter to allow for efficient perfusion through the leaky endothelial cells of the tumor. Prior to formation of the LPD’s the liposome formulation was 86 nm diameter, well below the maximum acceptable diameter (Table S1, below). The addition of DNA and peptide to the LPD was followed by re-concentration to 1.95mM, causing increase in particle size to 140 - 147 nm diameter. The polydispersity of the samples also increased slightly after LPD formation, reflecting the heterogenous formulation, but it is still within the ideal diameter for tumoral delivery.

To minimise particle aggregation and ensure the structural integrity of the LPD’s *in vivo*, a relatively high zeta potential (> ±40 mV) is required. Inclusion of the cationic lipids DOTMA and DODEG-4, accounting for 15% and 39% of the total lipid-cholesterol composition respectively, gave the initial liposome formulation stability, with a zeta potential of +45 mV. The subsequent addition of peptide and DNA did not substantially alter this value, yielding serum stable LPD’s, with potentials of between +41 mV (± 10.3 mV) and +57 mV (± 7.3 mV).

| **Liposome** | **DLS /nm (PDI)** | **Zeta pot. /mV** |
| --- | --- | --- |
|  |  |  |
| *In vivo (****1****)* | 85.7 (0.274) | 43.8 (±5.8) |
| *In vivo (****2****)* | 147.1 (0.355) | 40.6 (±10.3) |
| *In vivo (****3****)* | 140.2 (0.407) | 56.7 (±7.3) |

**Table S1. DLS and Zeta potential of liposome and LPD batches for *in vivo* injection;** (**1**) Liposome sample; (**2**) LPD formulated from Picchu-X plasmid and peptide **Y-I**; (**3**) LPD formulated from GFP plasmid and peptide **Y-I**.

Design and construction of Picchu-X. The Picchu-X sensor (YFP-CrkII-CFP) for reporting EGFR kinase activity was a kind gift from M. Matsuda (Osaka University, Japan) (X stands for the introduction of a KRas CAAX sequence that targets the biosensor to the cell membrane) ([4-6](#_ENREF_4)). The sensing part (CrkII-based) was excised and placed into previously described Raichu-Rac 1011-X sensor ([7](#_ENREF_7)). The construct was verified by Sanger sequencing ([5](#_ENREF_5)). The Picchu-X-GFP control (without mRFP1) was constructed by excision of mRFP1 protein encoding sequence.

**qRT-PCR and primers.** mRNA was extracted from cell lysates using the Absolutely RNA Miniprep Kit (Aligent Technologies). mRNA expression was analysed by quantitative real-time PCR using the EXPRESS One-step Superscript® qRT-PCR kit (Thermo Fisher Scientific), using assay on demand (Thermo Fisher Scientific) primers and 5’-FAM™ and 3’-TAMRA™ labelled probes (Integrated DNA Technologies): Nos2 Mm00440502_m1 and Tbp Mm01277045_m1. qRT-PCR was performed using an ABI 7900HT Fast Real Time PCR instrument (Thermo Fisher Scientific).

The epifluorescence intensity image was obtained with an overall scan of the whole well in a 96-well plate using a motorised microscope system. ImageJ software was used for the analysis of normalised-pixel count (Cy5/UV for liposomal uptake and eGFP/UV for biosensor expression).

**Single-photon emission computed tomography (SPECT)/CT imaging (instrumentation setting)**. SPECT was acquired prior to CT, using an exposure time of 1200 s, obtained over 24 projections (50s per projection), a 4-head scanner with a 4 x 9 array of 1 mm pinhole apertures in helical scan mode with a total acquisition time of 30 min. CT images were acquired using a 55 kVP X-ray source, 1000 ms projection exposure time in 180 projections at pitch of 1 with an acquisition time of 9 min. SPECT images were reconstructed in a 256 x 256 matrix using HiSPECT (ScivisGmbH, Bioscan), whereas, CT images were reconstructed in a 352 x 352 matrix using proprietary Bioscan InVivoQuant (Version 1.23 Bioscan, USA) software. Images were fused and correlated using InVivoQuant. 3D regions of interest were drawn using InVivoQuant over the main organs of clearance at each time point to assess the rate of clearance.

**Figure S9.** Modulation of ligand-enhanced EGFR phosphorylation by pharmacological inhibition (A and B) or gene knockdown (C). A) Long-term inhibition of EGFR phosphorylation by Mo-IPQA. HCC19554 cells were treated with Mo-IPQA (10 μM) for indicated time periods and level of phosphorylation of EGFR was detected. B) Effect of Mo-IPQA on phosphorylation of EGFR in HCC1954 and MDA-MB-231 cell lines. Cells were treated with indicated concentrations of Mo-IPQA for 1 hour before stimulation with EGF (100ng/mL, 30 min). Incomplete inhibition in MD-MB231 cells at highest dose of the inhibitor was observed, compared to HCC1954 cells. C) HCC1954 cells transfected with siRNA for EGFR family members, none related proteins and Picchu-X biosensor; cells were either untreated or stimulated with EGF (100 ng/mL, 30 min). The increase (enhancement) of FRET efficiency by EGF stimulation (compared with untreated control) is plotted for each siRNA. * indicates p <0.05 for the ligand-enhanced increase in FRET efficiency.


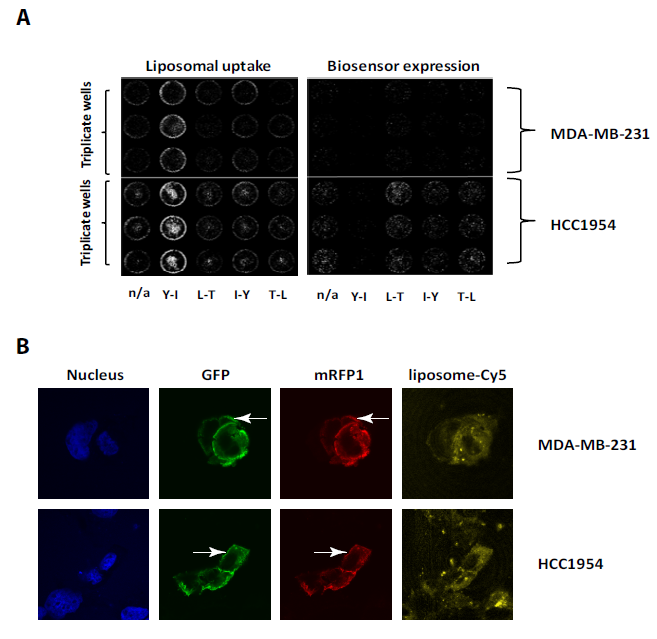


**Figure S10**. A) Effect of different targeting peptides on transfection (liposomal uptake, Cy5) and expression of Picchu-X (GFP) in HCC1954 and MDA-MB231 cell lines. N/A – peptide K_16_ (no targeting) used, **Y-I** = K_16_-RVRR-YHWYGYTPQNVI, **L-T**= K_16_-RVRR-LARLLT. **I-Y** = YHWYGYTPQNVI-RVRR-K_16_, **T-L** = LARLLT-RVRR-K_16_. B) Localization of Picchu-X biosensor to plasma membrane (white arrows) after transfection with lipopolyplex complex.


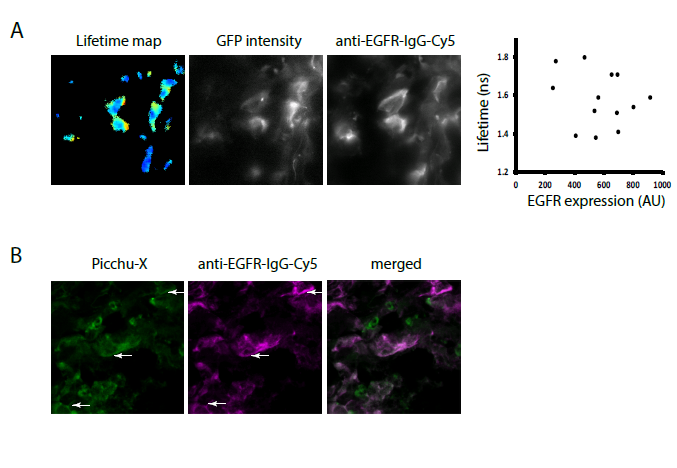

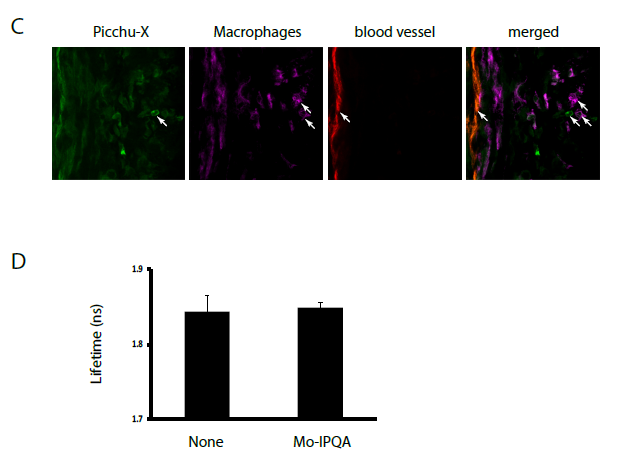


**Figure S11.** Relationship between Picchu-X biosensor response and EGFR expression

A) HCC1954 tumor tissue was sectioned and stained with a rabbit anti-EGFR antibody followed by a goat-anti-rabbit IgG-Cy5 conjugate. Average intensity for Cy5 (EGFR expression) was measured for each cell in the image and plotted against measured lifetime of GFP. No correlation between EGFR expression (averaged for the Picchu-X expressing cells – included in a mask created using GFP intensity image, in each field of view) and lifetime of GFP (averaged for each field of view) was found. Scatter plot for N = 9 fields of view is presented.

B) Representative confocal image showing colocalisation of Picchu-X biosensor and EGFR on plasma membrane (white arrors).

C) Representative confocal image showing the location of Picchu X-expressing cancer cell in relation to macrophages (F4/80, labelled with Cy5 antibody conjugate)and blood vessel (CD31, labelled with Cy3 antibody conjugate).

D) Lifetime of Picchu-X-GFP (altered molecule of biosensor with removed mRFP1 protein) in tissue from control and Mo-IPQA treated mice (N>7).

**
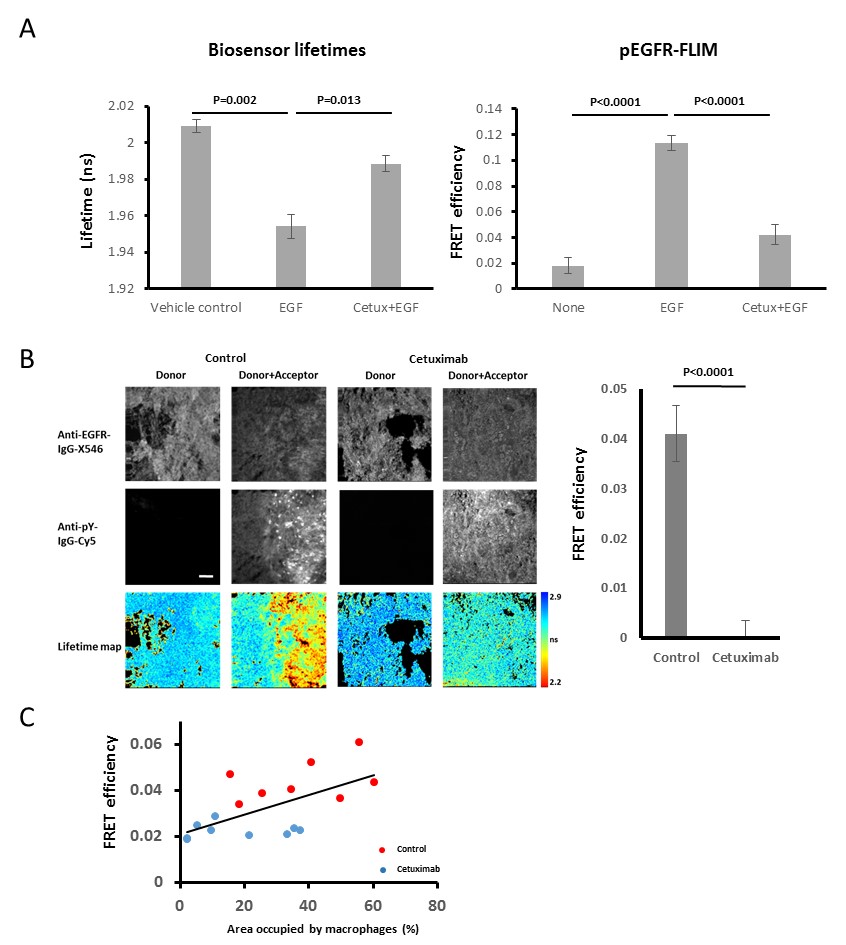
**

**Figure S12.** **EGFR activity imaging by Picchu-FLIM in LIM1215 cells and antibody based pEGFR FLIM assay in xenografts.**

A) Comparison between measurements of EGFR activity using Picchu FLIM or antibody based pEGFR FLIM assay. LIM1215 cells were left untreated or stimulated with EGF (100ng/ml) with or without a prior 1h treatment with cetuximab (100uM). For pEGFR FLIM, cells were fixed and stained with anti-EGFR-IgG-X546 (Alexa Fluor546) only (donor) or together with anti-pTyr-DL650 (donor + acceptor). Images of GFP and AlexaFluor546 of the same field of view were taken for analysis. Data expressed as mean ± SEM (N=6).

B) Mice were inoculated with LIM1215 tumor cells and after 2 weeks were treated with 20mg/kg cetuximab (intraperitoneally) for a further two weeks. FLIM measurements were obtained from the excised tumors, which were stained with anti-EGFR-IgG-X546 and with anti-pTyr-IgG-DL650. Activation of EGFR (increase in yellow-red pixels, scale bar = 50μm) was significantly inhibited by treatment with cetuximab. FRET efficiency (graph) was calculated according to: 1-(τ(donor+acceptor)/ τ(donor)), where τ(donor) is lifetime measured in images stained only with anti-EGFR-IgG-X546 antibody, and τ(donor+acceptor) is lifetime measured in images stained with anti-EGFR-IgG-X546 and anti-pTyr-IgG-DL650 antibodies. * P<0.0001, N=10 images per group

C) LIM1215 xenograft tissue sections were fixed and stained with anti-EGFR-IgG-X546 only or together with anti-pTyr-DL650 and with rat anti-F4/80 mAb (anti-macrophage IgG) + goat anti-rat IgG-Alexa647. Graph shows a significant correlation between FRET efficiency (EGFR activation) and the degree of macrophage infiltration in the same field of view (black line). Overall, every percent increase of area occupied by macrophages corresponded to 0.0004 (95% CI: 0.0001 to 0.0007, **P=0.01**) increase in FRET efficiency. There is no statistically significant interaction when we included the product of %mac and Cetuximab (P_interaction_=**0.35**).


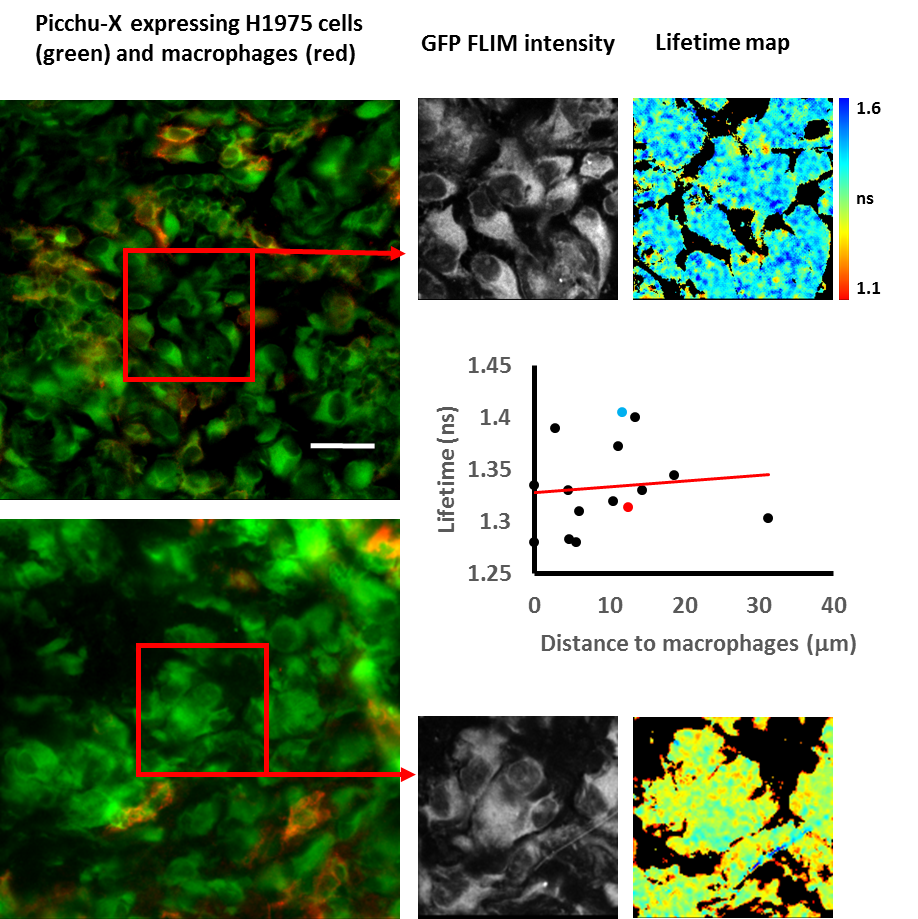
**Figure S13. EGFR activity imaging by Picchu-FLIM in H1975 xenografts.** Merged images of GFP (green) and Cy5 (red) fluorescent images show the localization of H1975 tumor cells (expressing the GFP- and mRFP- containing Picchu-X sensor) and macrophages (stained with a rat anti-F4/80 mAb + goat anti-rat IgG-Alexa647) in xenograft tissues. The inset (red square area) indicates the region of interest for the FLIM image taken with a multiphoton laser scanning system, as previously published[^10^](#_ENREF_10). Scale bar = 25 μm. The graph illustrates the relationship between EGFR activity (low GFP fluorescence lifetime of the Picchu-X probe corresponds to high EGFR activity, depicted as red pixels in the pseudocolor cell image) and distance between cancer cell and the nearest macrophage. The red and blue dots refer to the mean FRET efficencies measured in regions of interest in the lower and upper panels, respectively. No significant correlation (F=0.16, P=0.69) was seen *c.f.* the significant correlation between high EGFR activity in tumor cells and macrophage-tumor cell proximity, in HCC1954 breast xenografts which express WT EGFR (original Figure 5D).

**References**

1. Hurley CA, Wong JB, Hailes HC, Tabor AB. Asymmetric synthesis of dialkyloxy-3-alkylammonium cationic lipids. The Journal of organic chemistry. 2004;69:980-3.

2. Priet S, Zlatev I, Barvik I, Geerts K, Leyssen P, Neyts J, et al. 3'-Deoxy phosphoramidate dinucleosides as improved inhibitors of hepatitis C virus subgenomic replicon and NS5B polymerase activity. J Med Chem. 2010;53:6608-17.

3. Pal A, Glekas A, Doubrovin M, Balatoni J, Namavari M, Beresten T, et al. Molecular imaging of EGFR kinase activity in tumors with 124I-labeled small molecular tracer and positron emission tomography. Molecular imaging and biology : MIB : the official publication of the Academy of Molecular Imaging. 2006;8:262-77.

4. Aoki K, Kiyokawa E, Nakamura T, Matsuda M. Visualization of growth signal transduction cascades in living cells with genetically encoded probes based on Forster resonance energy transfer. Philosophical transactions of the Royal Society of London Series B, Biological sciences. 2008;363:2143-51.

5. Itoh RE, Kurokawa K, Fujioka A, Sharma A, Mayer BJ, Matsuda M. A FRET-based probe for epidermal growth factor receptor bound non-covalently to a pair of synthetic amphipathic helixes. Experimental cell research. 2005;307:142-52.

6. Kurokawa K, Mochizuki N, Ohba Y, Mizuno H, Miyawaki A, Matsuda M. A pair of fluorescent resonance energy transfer-based probes for tyrosine phosphorylation of the CrkII adaptor protein in vivo. J Biol Chem. 2001;276:31305-10.

7. Vega FM, Fruhwirth G, Ng T, Ridley AJ. RhoA and RhoC have distinct roles in migration and invasion by acting through different targets. The Journal of cell biology. 2011;193:655-65.
